# Dimension reduction of thermoelectric properties using barycentric polynomial interpolation at Chebyshev nodes

**DOI:** 10.1038/s41598-020-70320-7

**Published:** 2020-08-10

**Authors:** Jaywan Chung, Byungki Ryu, SuDong Park

**Affiliations:** Energy Conversion Research Center, Korea Electrotechnology Research Institute (KERI), Changwon-si, Gyeongsangnam-do, 51543 Republic of Korea

**Keywords:** Applied mathematics, Scientific data, Thermoelectric devices and materials

## Abstract

The thermoelectric properties (TEPs), consisting of Seebeck coefficient, electrical resistivity and thermal conductivity, are infinite-dimensional vectors because they depend on temperature. Accordingly, a projection of them into a finite-dimensional space is inevitable for use in computers. In this paper, as a dimension reduction method, we validate the use of high-order polynomial interpolation of TEPs at Chebyshev nodes of the second kind. To avoid the numerical instability of high order Lagrange polynomial interpolation, we use the barycentric formula. The numerical tests on 276 sets of published TEPs show at least 8 nodes are recommended to preserve the positivity of electrical resistivity and thermal conductivity. With 11 nodes, the interpolation causes about 2% error in TEPs and only 0.4% error in thermoelectric generator module performance. The robustness of our method against noise in TEPs is also tested; as the relative error caused by the interpolation of TEPs is almost the same as the relative size of noise, the interpolation does not cause unnecessarily high oscillation at unsampled points. The accuracy and robustness of the interpolation indicate digitizing infinite-dimensional univariate material data is practicable with tens or less data points. Furthermore, since a large interpolation error comes from a drastic change of data, the interpolation can be used to detect an anomaly such as a phase transition.

## Introduction

A relationship between two physical properties is usually represented by a function of a real variable. If an analytic formula of the function is unavailable, the relationship cannot be completely described by a finite number of values since the function is an infinite-dimensional vector. Sharing the relationship for use in computers is also demanding because computers can handle only a finite number of values. The best remaining option is to project the infinite-dimensional data into a finite-dimensional space, and recover the data. As an example, physical properties from observations are given only at finite data points, and at other infinite data points, a presumption is made. The physical properties are often estimated by regression or linear interpolation. But regression does not preserve the observed data since it is a compromise between a regression model and the observed data. Furthermore, achieving high accuracy by regression is difficult because it requires the correct choice of a regression model. If the observed data is unreliable, regression may be preferable because a regression model alleviates anomalies. But here we assume the observed data is reliable and valuable. This is particularly true when the data is obtained from numerical simulations. On the other hand, linear interpolation is a simple method to find a curve preserving the observed data, but the curve is no longer differentiable. If one demands a smooth curve preserving the observed data, another approach is necessary. In this paper, we demonstrate an interpolation method to reconstruct a smooth curve from a finite number of data points, exemplified by thermoelectric material properties.

The thermoelectric effect^1^, a direct and reversible energy conversion between electricity and heat, is governed by three thermoelectric material properties (TEPs): Seebeck coefficient $\alpha$, electrical resistivity $\rho$, and thermal conductivity $\kappa$. The performance parameters of thermoelectric power generation modules such as power output and efficiency are numerically computable with a given set of TEPs^2^. However, since all the TEPs depend on temperature *T*, they are infinite-dimensional vectors as functions of a real variable $T{:}\,\alpha =\alpha (T), \rho =\rho (T)$, and $\kappa =\kappa (T)$. The infinite dimensionality of the TEPs hinders the numerical computations since computers can accept only a finite number of values as input. Therefore it is unavoidable to describe the TEPs with a finite number of values, i.e., to project the infinite-dimensional material properties into a finite-dimensional space and reconstruct them.

One way to reduce the dimension is to extract TEP values at a finite number of temperature values. Then the full TEP curves are reconstructed by interpolation which preserves the raw data. Suppose $n+1$ sample values $f_j, j=0,1,\ldots ,n,$ of a TEP are extracted at $n+1$ distinct temperature values $T_j, j=0,1,\ldots ,n$ where $T_j$’s are strictly increasing: $T_0< T_1< \cdots < T_n$. Among the many interpolation methods, here we focus on polynomial interpolation because it is computationally cheap, and the derivatives and integrals of polynomials are directly obtainable. The ease of differentiation can help to calculate significant transport properties such as the effective masses of electrons and holes. A well-known formula for polynomial interpolation is the Lagrange formula:

1 $$\begin{aligned} p_n(T) = \sum _{j=0}^n f_j \ell _j(T) \end{aligned}$$

where $\ell _j$ is the Lagrange polynomial

2 $$\begin{aligned} \ell _j(T) := \frac{\prod _{k=0, k\not =j}^n (T-T_k)}{\prod _{k=0, k\not = j}^n(T_j-T_k)} \end{aligned}$$

which satisfies $\ell _j(T_j)=1$ and $\ell _j(T_k)=0$ for $j\not = k$. The subscript *n* of the $p_n(T)$ denotes the degree of the polynomial.

A popular choice of $T_j$ is *equidistant nodes*:

$$\begin{aligned} T_j := \frac{T_n-T_0}{n}j + T_0, \quad j=0,1,\ldots ,n. \end{aligned}$$

**Figure 1:**
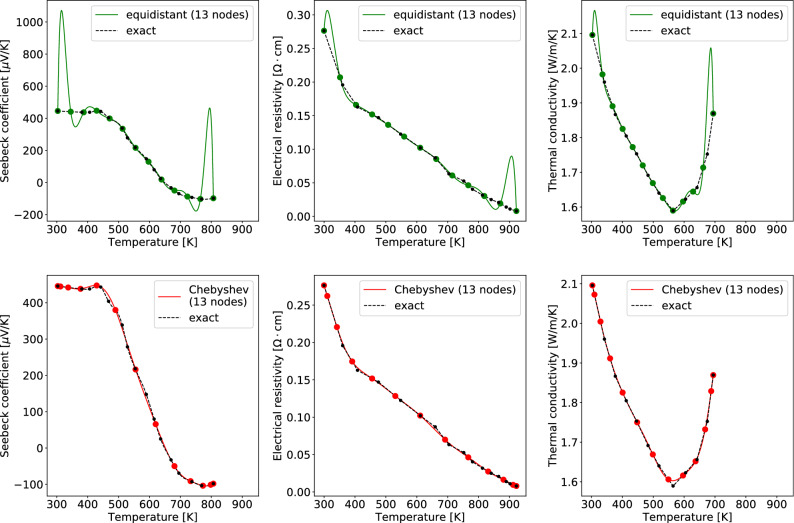
The polynomial interpolation of a set of TEPs in^4^ at (top) equidistant nodes and (bottom) Chebyshev nodes: (left) Seebeck coefficient $\alpha (T)$, (center) electrical resistivity $\rho (T)$, (right) thermal conductivity $\kappa (T)$. The black dots denote the raw data points. The exact curve is assumed to be a quadratic spline for Seebeck coefficient and a linear spline for electrical resistivity and thermal conductivity. The green dots (the red dots) denote data points at 13 equidistant nodes (Chebyshev nodes) resampled from the exact curve.

However, the polynomial interpolation at equidistant nodes generates superfluous oscillations near the boundary of the interval $[T_0, T_n]$ for large *n*’s and even diverges as $n\rightarrow \infty$, as Runge^3^ first proved with the function $f(x)=(1+x^2)^{-1}$, $x \in [-5,5]$. The Runge’s phenomenon arises naturally for many continuous curves. As an example, consider a Ag-doped $\hbox {Mg}_2\hbox {Si}_{0.6}\hbox {Ge}_{0.4}$ thermoelectric material in^4^. The top of Fig. 1 shows the polynomial interpolation of the TEPs highly deviates from the exact curve near the boundaries of the temperature intervals.

To alleviate the Runge’s phenomenon, the choice of sample nodes $T_j$ is critical; the density of sample points should follow an asymptotic density proportional to $(1-x^2)^{-1/2}$ when the interval of *x* is $[-1,1]$. Hence the density should be higher near the boundaries of the interval than the inside. One of such a choice is *Chebyshev nodes* of the second kind:

3 $$\begin{aligned} T_j := \frac{T_0-T_n}{2} \cos \frac{j\pi }{n} + \frac{T_0+T_n}{2}, \quad j=0, 1, \ldots , n. \end{aligned}$$

A mathematical theory^5^ shows the Runge’s phenomenon would not be severe under the use of the Chebyshev nodes. The bottom of Fig. 1 shows that the polynomial interpolation at the Chebyshev nodes gives a substantially better result than the top of Fig. 1, overcoming the Runge’s phenomenon.

But still there is a computational issue in the Lagrange formula. When *n* is large, the direct computation of the Lagrange formula (1) is numerically unstable due to the high degree of the Lagrange polynomials (2); the numerator of $\ell _j(T)$ essentially contains the $T^n$ term so even with a moderate *T*, the numerator becomes too large to evaluate for large *n*. We use the *barycentric formula* of polynomial interpolation^6,7^ as a numerically stable algorithm.

In this paper, using experimental thermoelectric data, we validate the use of the barycentric polynomial interpolation at the Chebyshev nodes of the second kind as an accurate dimension reduction method for thermoelectric material property curves. The interpolation is tested for 276 sets of TEPs acquired from published papers. Information on the TEP dataset can be found in Supplementary Information (SI). In the following section, the barycentric formula and its derivation are given. In subsequent sections, the accuracy of the interpolation on TEPs and module performance (figure of merit *zT*, power density, and efficiency) is studied. Then the effect of noise on the accuracy is tested. We conclude that the interpolation is accurate and robust for continuous TEPs, allowing its further application into various curves of scientific data.

## Methods

### Barycentric formula of polynomial interpolation

Since the barycentric formula has not been emphasized in elementary numerical analysis, here we include its derivation by following^8^. Let us define $\ell (T) := \prod _{k=0}^n (T-T_k)$ and the barycentric weights by

4 $$\begin{aligned} w_j := \frac{1}{\prod _{k=0,k\not =j}^n (T_j-T_k)}. \end{aligned}$$

Then obviously $\ell _j(T) = \ell (T) \frac{w_j}{T-T_j}$ from (2). Hence from the Lagrange formula (1) we have

5 $$\begin{aligned} p_n(T) = \ell (T) \sum _{j=0}^n \frac{w_j}{T-T_j} f_j. \end{aligned}$$

If the constant function 1 is interpolated, all the $f_j$’s are 1 and the right-hand side of (5) should be 1. Hence the modified Lagrange formula (5) yields

$$\begin{aligned} 1 = \ell (T) \sum _{j=0}^n \frac{w_j}{T-T_j}. \end{aligned}$$

Inserting this relation into (5), we have the *barycentric formula*:

6 $$\begin{aligned} p_n(T) = \frac{\displaystyle \sum _{j=0}^n \frac{w_j}{T-T_j} f_j}{\displaystyle \sum _{j=0}^n \frac{w_j}{T-T_j}}. \end{aligned}$$

Since the numerator and denominator in (6) have the same barycentric weights $w_j$, any scaling of (4) can be used instead. For equidistant nodes, the barycentric weights can be explicitly computed by $$w_j = (-1)^j \left( {\begin{array}{c}n\\ j\end{array}}\right)$$ with a proper scaling^8^, where $$\left( {\begin{array}{c}n\\ j\end{array}}\right)$$ is the binomial coefficient. For the Chebyshev nodes (3),

7 $$\begin{aligned} w_j = {\left\{ \begin{array}{ll} (-1)^j \frac{1}{2} &{} \text {if }j=0\text { or }j=n,\\ (-1)^j &{} \text {otherwise,} \end{array}\right. } \end{aligned}$$

with a proper scaling^9^. This simplicity of $w_j$’s makes the choice of the Chebyshev nodes (3) particularly intriguing among other choices of nodes. The barycentric polynomial interpolation (6) with (7) is explicit, hence its computational cost is cheap.

Due to the singular term $1/(T-T_j)$ in (6), the barycentric formula (6) is not defined at $T=T_j$ and need to be specially treated as the sample value $f_j$. However, when $T \simeq T_j$, because the numerator and the denominator have the same singular terms $1/(T-T_j)$, the inaccuracies due to the singular terms may cancel out^8^. If we use the Chebyshev nodes (3), the barycentric formula is indeed numerically forward stable^10^ and no severe inaccuracy arises at $T \simeq T_j$.

The derivative of the barycentric formula (6) can be written in the same form. Using the barycentric representation of $\ell _j(T)$, we can show^8^ that

$$\begin{aligned} \ell _j'(T_i) = {\left\{ \begin{array}{ll} \displaystyle \frac{w_j/w_i}{T_i-T_j} &{} \text {if }j \not = i,\\ \displaystyle -\sum _{k\not = i} \frac{w_k/w_i}{T_i-T_k} &{} \text {if }j=i. \end{array}\right. } \end{aligned}$$

From the Lagrange formula (1), $p_n'(T_i) = \sum _{j=0}^n f_j \ell _j'(T_i)$ hence

8 $$\begin{aligned} p_n'(T_i) =\sum _{j\not = i} \frac{(w_j/w_i)(f_j-f_i)}{T_i-T_j}. \end{aligned}$$

Considering the $p_n'(T_i)$ above as a new sample value at $T_i$, we have the barycentric formula of $p_n'(T)$ just replacing $f_i$ in (6) by $p_n'(T_i)$ in (8):

$$\begin{aligned} p_n'(T) = \frac{\displaystyle \sum _{j=0}^n \frac{w_j}{T-T_j} p_n'(T_j)}{\displaystyle \sum _{j=0}^n \frac{w_j}{T-T_j}}. \end{aligned}$$

## Results

### Accuracy of interpolation

We test the barycentric polynomial interpolation (6) at the Chebyshev nodes (3) by reconstructing 276 sets of TEPs from published papers. The list of the papers are given in the SI. The TEP dataset was previously used to validate a theory of thermoelectric conversion efficiency in^11,12^.

To assess the accuracy of interpolating curves, an exact curve should be known but this is not possible since the determination of the exact curve requires an infinite (uncountable) number of measurements. Hence we *assume* that Seebeck coefficient curve $\alpha (T)$ is given by a second-order spline (a spline is a piecewise polynomial; see, e.g.,^13^), and electrical resistivity $\rho (T)$ and thermal conductivity $\kappa (T)$ curves are given by first-order splines (i.e., piecewise linear curves). With this assumption, only one exact curve is obtained for each TEP from the raw data points. We use a second-order spline for $\alpha$ because our evaluation of thermoelectric module performance requires the temperature derivative of Seebeck coefficient $\alpha '(T)=\frac{d\alpha }{dT}(T)$; this point will be clear in the next section. We use first-order splines for $\rho$ and $\kappa$ to secure the strict positivity of $\rho$ and $\kappa$; higher-order splines can give unphysical properties of zero or negative $\rho$ and $\kappa$ due to superfluous oscillations. Also note that the choice of the piecewise linear exact curve makes polynomial interpolation even harder, compared to the choice of higher-order splines; it would not be an easy task for smooth polynomials to imitate non-differentiable piecewise linear curves.

**Figure 2:**
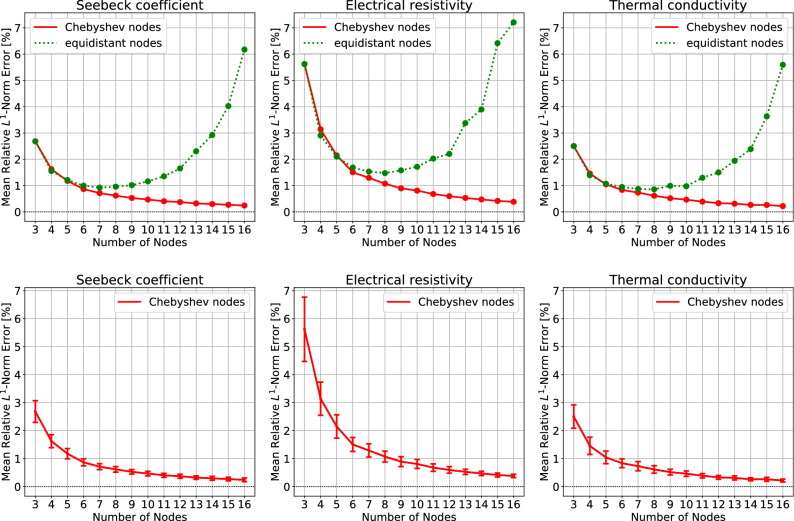
The mean relative $L^1$-norm error arising from the barycentric polynomial interpolation of TEPs, averaged over 276 TEPs. The interpolated TEP curves are compared to the exact TEP curves. (top) Chebyshev nodes vs. equidistant nodes, (bottom) Chebyshev-node case only, with error bars. The error bars indicate the 95% confidence intervals (CIs) for population mean. The CIs for equidistant nodes are omitted here and given in the SI because they are too large to draw.

The top of Fig. 2 shows the superiority of Chebyshev nodes over equidistant nodes. In the figure, the relative error is measured by the $L^1$-norm:

$$\begin{aligned} \frac{\Vert f-{\hat{f}}\Vert _1}{\Vert f\Vert _1}, \quad \Vert f\Vert _1 := \int _{T_0}^{T_n} |f(T)|\,dT \end{aligned}$$

where *f* is an exact function and ${\hat{f}}$ is an interpolating function. As the number of nodes *n* increases, the error of the interpolation at Chebyshev nodes consistently decreases. With 11 Chebyshev nodes one may expect relative $L^1$-norm errors of 0.5% for $\alpha$ and $\kappa$, and 1% for $\rho$; see the bottom of Fig. 2. Meanwhile, the error of the interpolation at equidistant nodes significantly increases for large *n*’s: the error is at the minimum with 7 nodes and exceeds 6% with 16 nodes.

**Figure 3:**
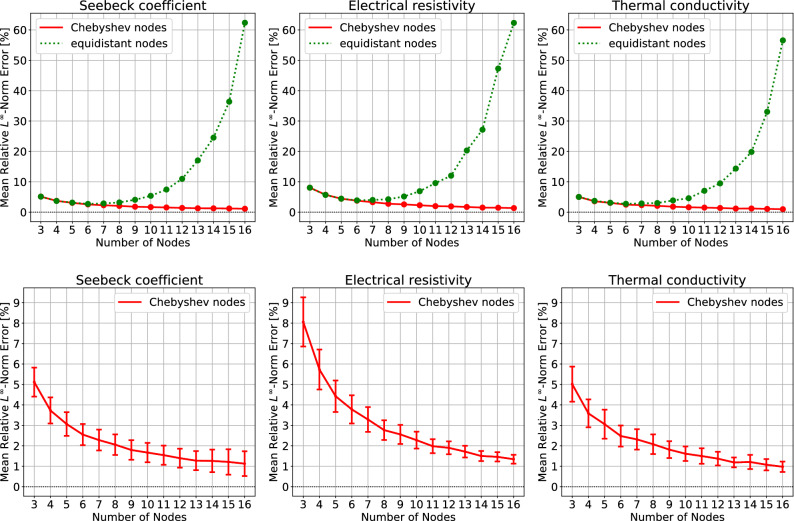
The mean relative $L^\infty$-norm error in the barycentric polynomial interpolation of TEPs, averaged over 276 TEPs. (top) Chebyshev nodes vs. equidistant nodes, (bottom) Chebyshev-node case only, with error bars. The error bars indicate the 95% CIs for population mean. The omitted CIs for equidistant nodes are given in the SI.

As shown in the top of Fig. 3, the superiority of Chebyshev nodes is more apparent if the relative $L^\infty$-norm

$$\begin{aligned} \frac{\Vert f-{\hat{f}}\Vert _\infty }{\Vert f\Vert _\infty }, \quad \Vert f\Vert _\infty := \max _{T \in [T_0,T_n]} |f(T)| \end{aligned}$$

is considered. The error for equidistant nodes grows serious and exceeds 50% with 16 nodes, while the error for Chebyshev nodes consistently decreases. With 11 Chebyshev nodes, one may expect relative $L^\infty$-norm errors of 2% for $\alpha$ and $\kappa$, and 2.5% for $\rho$, as shown in the bottom of Fig. 3.

### Accuracy of module performance

Here we examine how much error in performance of thermoelectric modules is caused by the polynomial interpolation of $\alpha (T)$, $\rho (T)$ and $\kappa (T)$. We consider a single-material single-leg thermoelectric power generation module with the length *L* of $1 \,\mathrm {mm}$ and cross-sectional area *A* of $1 \,\mathrm {mm^2}$. Then the temperature distribution *T*(*x*) inside the module with a spatial coordinate $x \in [0,L]$ is given by the following second-order ordinary differential equation called the thermoelectric equation (for derivation, refer to^2^):

9 $$\begin{aligned} \frac{d}{dx}\Big (-\kappa (T)\frac{dT}{dx}\Big ) = \rho (T) J^2 - \frac{d\alpha }{dT}(T) \,T \frac{dT}{dx} J, \end{aligned}$$

where *J* is a given electric current density: $J=I/A$ for a given electric current *I*. We assume the thermoelectric module is under fixed temperatures at the boundaries: $T(0)=T_h$ and $T(L)=T_c$. The hot-side temperature $T_h$ and the cold-side temperature $T_c$ are chosen as the maximum and minimum temperature values in the TEP data where all the $\alpha (T), \rho (T)$ and $\kappa (T)$ are available. As before, the exact curves are assumed to be a second-order spline for $\alpha (T)$, and first-order splines for $\rho (T)$ and $\kappa (T)$. We avoided using a first-order spline for $\alpha (T)$ because the thermoelectric equation (9) contains the derivative of $\alpha (T)$; if $\alpha (T)$ is a first-order spline, then its derivative is discontinuous so the computation of a numerical solution of (9) becomes difficult.

The power *P* generated by the thermoelectric module is given by

$$\begin{aligned} P = I (V_{\mathrm {OC}} - IR) \end{aligned}$$

where the $V_{\mathrm {OC}}$ is the open-circuit voltage and *R* is the electrical resistance inside the module: $V_{\mathrm {OC}} = \int _{T_c}^{T_h}\alpha (T)\,dT$ and $R = \frac{1}{A}\int _0^L \rho (T(x))\,dx$. The energy conversion efficiency of the module is given by

$$\begin{aligned} \eta = \frac{P}{-\kappa (T_h)A\frac{dT}{dx}(0) + I \alpha (T_h) T_h}. \end{aligned}$$

Since the power and the efficiency depend on the given electric current *I*, we can maximize the power or efficiency by choosing a suitable *I*. Such maximum values are referred as the maximum power and maximum efficiency. Another popular performance parameter is the thermoelectric figure of merit *zT*. Here the *z* is defined by

10 $$\begin{aligned} z(T)=\frac{\alpha ^2(T)}{\rho (T)\kappa (T)}. \end{aligned}$$

The $zT_m := z \frac{T_h+T_c}{2}$ is proportional to the efficiency for temperature-*independent* TEPs^14^. Although the proportional relation is no longer valid for temperature-dependent TEPs^11,12,15–17^, the *zT* has been widely used to assess thermoelectric materials due to the conciseness of the formula. Because the *zT* depends on *T*, the maximum value of *zT* over $T \in [T_c,T_h]$ is often used for material evalulation.

**Figure 4:**
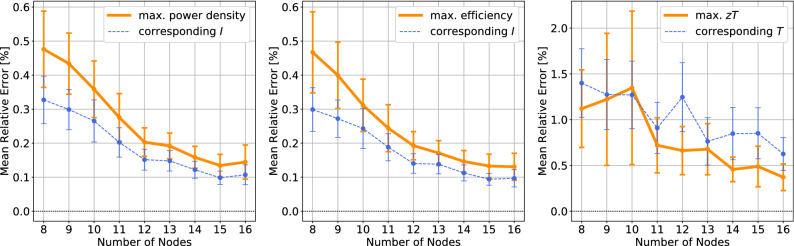
The mean relative error in three parameters of module performance, averaged over 276 TEPs: (left) maximum power density *P*/*A* and the corresponding electric current *I* where the maximum is attained, (center) maximum efficiency $\eta$ and the corresponding electric current *I* where the maximum is attained, (right) maximum *zT* and the corresponding temperature *T* where the maximum is attained. The error bars indicate the 95% CIs for population mean.

Here we consider three performance parameters of thermoelectric power generation modules: the maximum power density *P*/*A*, maximum efficiency $\eta$, and maximum *zT*. The errors in those parameters, caused by the interpolation, are given in Fig. 4. In the figure, 8 or more nodes are considered because fewer nodes did not guarantee the positivity of $\rho (T)$ for some of 276 TEPs. At least 8 nodes are recommended to secure the strict positivity of $\rho (T)$ and $\kappa (T)$. With 11 Chebyshev nodes, one may expect the relative error of 0.4% for the maximum power density, maximum efficiency, and the corresponding electric currents where the maximum values are attained. The low error is in accordance with the $L^1$-norm error in the interpolation of TEPs. This is not surprising because the solution *T*(*x*) of (9) is mainly affected by integrated quantities of TEPs rather than the TEP themselves; refer to an integral formulation of the thermoelectric equation in^11,12,15^. On the contrary, the errors in the maximum *zT* and the corresponding *T* show a higher error of 1%. Since the *zT* depends on the TEPs directly, the error is in accordance with the $L^\infty$-norm error in the interpolation of TEPs.

### Robustness on noise

We have assumed so far the values extracted at Chebyshev nodes are exact. If there is noise in the sampling, how much the accuracy of the interpolation is affected? To assess the robustness on the noise, we add random noise on the extracted value in (6) by replacing $f_j$ with

$$\begin{aligned} f_j + U\Big (-f_j \times \frac{p}{100}, +f_j \times \frac{p}{100}\Big ), \end{aligned}$$

where *U*(*a*, *b*) is the uniform random variable of which probability density function is given by $f_U(x) = \frac{1}{b-a}$ if $x \in (a,b)$ and $f_U(x)\equiv 0$ otherwise. Let us call this process $p\%$*noise* for convenience.

**Figure 5:**
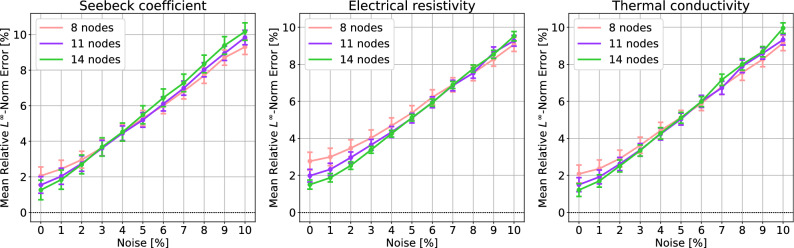
The mean relative $L^\infty$-norm error caused by the interpolation of noise-added TEPs, compared to the exact TEPs. The $L^\infty$-norm errors are averaged over 276 TEPs having noise in sampling at Chebyshev nodes. The error bars indicate the 95% CIs for population mean.

The Fig. 5 shows the error caused by the interpolation of TEPs linearly increases with the degree of noise. The percentage of the error is almost the same as the percentage of noise. This implies the interpolation does not cause unnecessarily high oscillation at unsampled points even there is sampling noise. A large number of nodes is slightly detrimental when there is a high degree of noise. This is because a high order polynomial needlessly struggles for interpolating the noise-added curves. But such degradation due to the choice of the number of nodes is less than 1%.

**Figure 6:**
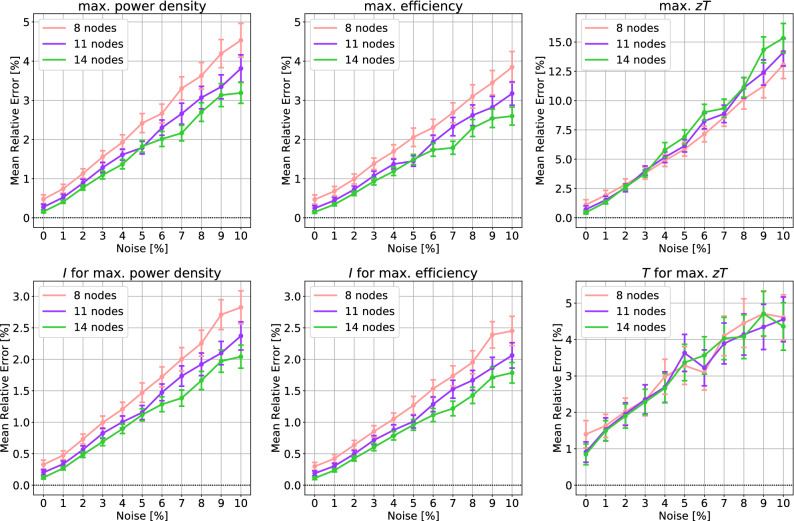
The mean relative error in module performance vs. degree of noise. The errors are averaged over 276 TEPs having noise in sampling at Chebyshev nodes. The error bars indicate the 95% CIs for population mean.

The Fig. 6 shows similar, linearly increasing error trends for module performance. The result is better for maximum power density and maximum efficiency; the percentage of error is below half of the percentage of noise. For example, with 11 Chebyshev nodes, the average relative error in maximum power density and maximum efficiency is below 2% under 5% noise. On the other hand, the result for the maximum *zT* is worse; the percentage of error is about one and a half of the percentage of noise. Also note that the error trends for the maximum *zT* are only valid when the interpolation preserves the positivity of $\rho$ and $\kappa$. If it does not due to the noise, the error soars because $\rho \times \kappa$ is the denominator of *z* as in (10). But negative $\rho$ and $\kappa$ are so unphysical and conspicuous that one would fix them by resampling. For that reason, we deliberately avoided such negative $\rho$ and $\kappa$ cases in the numerical simulation.

### Physical implication of small and large interpolation error cases

We have verified that tens or less Chebyshev nodes provide an accurate polynomial interpolation on average. But even a smaller number of nodes is enough for many TEP curves. For example, with 7 nodes, 45% of the interpolation results has less than 1.5% error in the relative $L^\infty$-norm, and 80% of the results has less than 4.8% error (see Table S17 in SI). Let us call such well-interpolated curves *normal* and the other curves *abnormal*.

**Figure 7:**
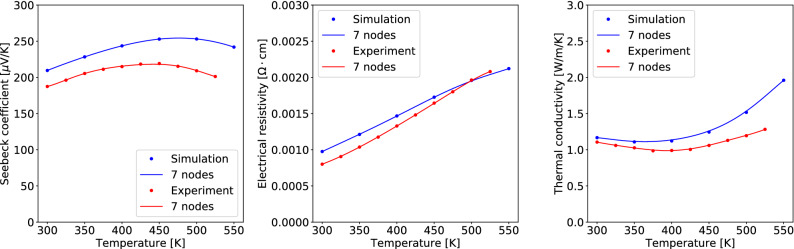
The 7-Chebyshev-node interpolation of measured and simulated TEPs for the nanostructured Bi-Sb-Te bulk alloys in^18^: (left) Seebeck coefficient, (center) electrical resistivity, (right) thermal conductivity. The red dots denote the raw data points in^18^. The blue dots denote the simulated TEPs using the Boltzmann transport equations with the relaxation time approximation within first-principles calculations. Both are well interpolated by 7 Chebyshev nodes. The details of the simulation method are given in SI.

As an example of a normal curve, Fig. 7 shows that the nanostructured Bi-Sb-Te bulk alloys^18^ have slowly varying behavior of measured TEP values in the observed temperature range smaller than 550K. The electron and phonon Boltzmann transport equations (BTE) with the relaxation time approximation (RTA) within first-principles calculations^19–21^ may predict the TEP curves of this material; refer to SI for the details of the method. The predicted curves in Fig. 7 not only follow the variation on temperature but also have a reliable size of TEP values. Within this semi-classical theory, the electron and phonon quasiparticles are responsible for the charge and heat transport in crystalline solids ^22^. Within the RTA, the interaction between fundamental particles and various imperfections lead to the particle scattering and enhanced resistivity^23–25^. Hence, when there is no phase transition by temperature change, the thermoelectric properties predicted by the BTE with RTA are smooth functions of temperature *T* because the charge and heat carrier densities as well as the relaxation time are smooth functions of *T*. The strong agreement between the measured and computed TEPs suggests that the nanostructured Bi-Sb-Te bulk alloys in^18^ have no material phase transition and their TEP curves are smooth.

**Figure 8:**
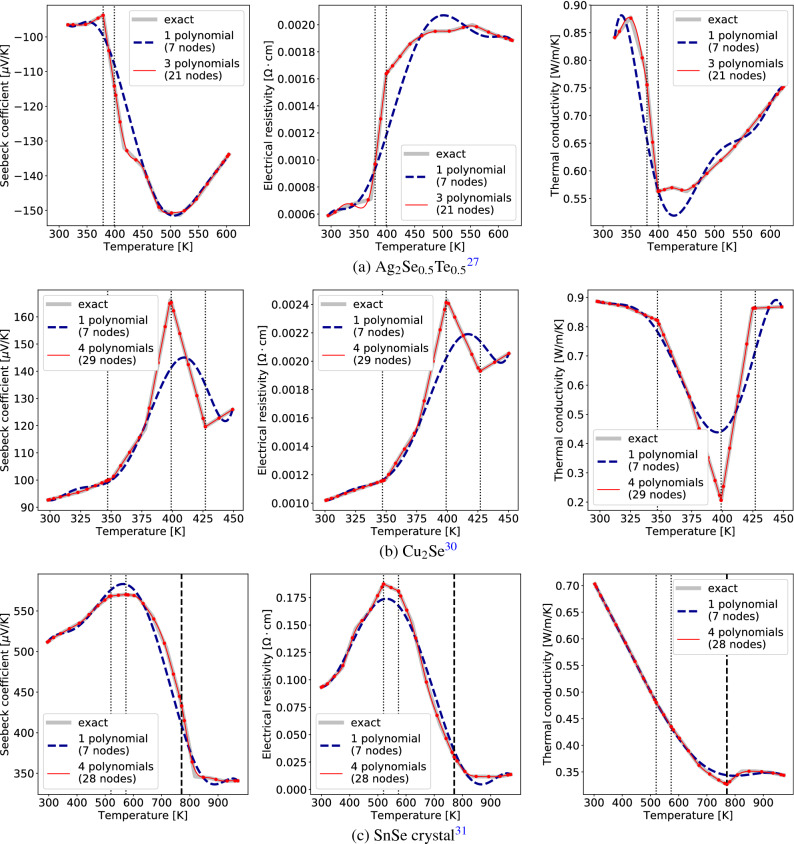
Examples of abnormal TEP curves in the dataset. As we suspect a phase transition, we assume all the exact curves (silver) are piecewise linear. The vertical lines indicate automatically detected abnormal temperature points. The dotted vertical lines are where the difference between the exact curve and the 7-Chebyshev-node polynomial interpolation (dark blue dashed line) is high. The dashed vertical line in (c) is where the difference between their derivatives is high. The detailed algorithm is given in SI. By dividing the temperature range with the detected temperature points and interpolating on each range, accurate interpolating curves (red) are found.

On the other hand, abnormal TEP curves have a large interpolation error with a small number of nodes because they undergo a drastic change with temperature. As a phase transition can be manifested through a drastic change or a discontinuity in TEP curves, temperature points where a large interpolation error occurs can be a phase transition temperature. Using this idea, we search for large interpolation error points to detect phase transition behaviors. Figure 8(a)–(c) show three examples of phase transition successfully detected by this method without using any domain knowledge. The detailed algorithm is given in SI.

In contrast to $\hbox {Bi}_2\hbox {Te}_3$-based thermoelectric materials, other tellurides can have phase transitions. One case is the $\hbox {Ag}_2$Te-based materials: at room temperature $\hbox {Ag}_2$Te has a low symmetric monoclinic phase and it transforms into a high temperature fcc phase above 417 K^26^. Consistent with this fact, our algorithm detects an abnormal temperature for $\hbox {Ag}_2\hbox {Se}_{0.5}\hbox {Te}_{0.5}$^27^ near 380 to 400 K; see Fig. 8(a). The drastic change of the TEP curves occurs in a narrow temperature range. It is argued that due to the alloying effect between $\hbox {Ag}_2\hbox {Te}$ and $\hbox {Ag}_2\hbox {Se}$, the phase transition seems to occur gradually in the narrow temperature range from 397 to 424 K^28,29^. Noteworthily, such phase-transition-induced abnormal transport behaviors are detected for all $\hbox {Ag}_2\hbox {Te}$-containing materials in our dataset.

Figure 8(b) shows the abnormal transport behavior of $\hbox {Cu}_2$Se^30^ known to exhibit a continuous phase transition. The continuous change of lattice angle in the temperature range from 340 to 410 K was confirmed by HTXRD data^30^. The key temperature points are well detected by our algorithm.

SnSe is one of the well-known thermoelectric materials having high *zT* of 2.6 along the *b*-direction in the single crystalline phase^31^. Figure 8(c) shows our data analysis detects several abnormal temperature points for this material. There are two important temperature points, $\sim$ 600 K and $\sim$ 800 K, where the slopes of Seebeck coefficient and electrical resistivity rapidly change. The detected temperature point in 600–800 K well coincides with the range under the thermally activated generation of hole charge carriers in SnSe reported in^32^. From density functional theory calculations, it is shown that the formation of acceptor Sn-vacancy is responsible for the increasing electrical conductivity with temperature^32^: $n_h = N \exp ( - E_{\mathrm {form}}/(kT))$ where $n_h$ is the hole carrier density, *N* is the site density of Sn, and $E_{\mathrm {form}}$ is the defect formation energy of Sn-vacancy. The next critical point is about 800 K. The origin of the derivative discontinuity at near $T = 800$ K is considered to be the phase transition from Pnma to Cmcm^31,32^.

### Mathematical interpretation of small and large interpolation error cases

We have observed that many TEP curves are accurately interpolated with even a small number of Chebyshev nodes, and a large interpolation may indicate a drastic change in the curves due to a phase transition. There is a mathematical basis for why the polynomial interpolation can perform well for many TEPs. For a given *continuous* function on a closed interval, there is a polynomial arbitrarily close to the given function in the $L^\infty$-norm, by the Stone-Weierstrass theorem (see, e.g., Theorem 7.26 in ^33^). The issue is to find such a polynomial under a limited number of sampling nodes, and our approach here is the sampling at Chebyshev nodes.

On the other hand, for a *discontinuous* function, there is no reason for a polynomial interpolation to work. Suppose *f*(*T*) is a real-valued, $(n+1)$-times continuously differentiable function on a closed interval $[T_0, T_n]$. Then by Taylors’ theorem (see, e.g., Theorem 5.15 in ^33^), the interpolating polynomial $p_n(T)$ of *f*(*T*) at $n+1$ nodes $\{T_j\}_{j=0}^{n}$ satisfies

$$\begin{aligned} f(T) - p_n(T) = \frac{f^{(n+1)}(\xi (T))}{(n+1)!} \prod _{j=0}^n (T-T_j), \end{aligned}$$

where $\xi (T) \in (T_0, T_n)$ is some value depending on *T*. If $\{T_j\}_{j=0}^{n}$ is the sequence of Chebyshev nodes of the second kind in (3), the normalized variable $s := \frac{T-T_0}{T_n-T_0}$ gives

$$\begin{aligned} f(T) - p_n(T) = \frac{{\hat{f}}^{(n+1)}({\hat{\xi }}(s))}{(n+1)!} \prod _{j=0}^n \Big (s - \frac{1}{2}\Big (1-\cos \frac{j \pi }{n}\Big )\Big ), \end{aligned}$$

where ${\hat{f}}(s) := f(T)$ and ${\hat{\xi }}(s) := \xi (T)$. The right-hand side shows the error depends on the normalized highest-order derivative ${\hat{f}}^{(n+1)}$. This explains why a drastically changing or discontinuous curve ruins the interpolation. Also note that the error does not depend on the size of the interval $T_n-T_0$ as long as the normalized highest-order derivative is the same. Hence the error is affected by the oscillation shape of the curves rather than the size of the interval. If the function *f* is continuous, there is a high-order polynomial arbitrary close to the *f*. As a polynomial has a high-order derivative, our Chebyshev-node interpolation becomes close to this polynomial as the number of nodes becomes large. Therefore by using more nodes, one can accurately recover the continuous function *f*. But for a discontinuous function, the discontinuity is not treatable with a high-order polynomial.

A simple method to handle a drastically changing or discontinuous curve is to first spot the problematic points and to apply our interpolation method in each interval where the curve is continuous and midly changing. Some examples of this approach are given in Fig. 8(a)–(c); the interpolation consisting of multiple polynomials (red lines) are noticeably improved over the single-polynomial interpolation (dark blue dashed lines). The problematic points used there is the previously found abnormal temperature points.

## Conclusion

To reduce the infinite dimension of TEPs into a finite dimension, we propose the use of high-order polynomial interpolation at Chebyshev nodes of the second kind. To evaluate polynomials in a numerically stable way, the barycentric formula is used. The tests on 276 sets of published TEPs show our interpolation method is accurate, and robust on noise. For example, with 11 Chebyshev nodes, the error caused by the interpolation is about 2% for TEPs and 0.4% for module performance parameters of maximum power density and maximum efficiency. Even if there is noise in sampling, the interpolation does not cause any further error beyond the degree of noise.

While a small number of nodes is enough for many TEP curves, drastically changing or discontinuous curves can have a high interpolation error. A simple remedy to enhance the accuracy is to confine the temperature range to where the set of TEPs is continuous and midly changing. Meanwhile, as the drastic change may indicate a phase transition, the interpolation method can be used to detect an unidentified phase transition.

Since our polynomial interpolation method is robust on noise and presumes no physical model, it would perform well for various curves other than TEPs. The method projects infinite-dimensional vectors acquired from scientific experiments into a finite-dimensional space. Then a smooth, raw-data-preserving curve is efficiently constructed using the barycentric formula. Our empirical validation shows that the dimension reduction method allows digitizing infinite-dimensional univariate material data, with tens or less data points. Hence the method enables using the infinite-dimensional data for material informatics and machine learning.

## Computational method

All the numerical computations in this paper are performed using the python programming language (version 3.6). The thermoelectric equation (9) with the Dirichlet boundary condition is solved by the fourth-order collocation method implemented in the solve_bvp function^34^ of SciPy library (version 1.2.1). The maximum power density and maximum efficiency are found by the Brent-Dekker optimization method implemented in the minimize_scalar function^35^ of the SciPy library.

## Supplementary information

Supplementary information

## Data Availability

The data generated during the current study is available in the GitHub repository https://github.com/jaywan-chung/tep-chebyshev. More information on the TEP dataset is given in SI. The numeric values of the confidence intervals and standard deviations in Fig. 2, 3, 4, 5 and 6 are given in SI.

## Code availability

The code used to generate data and figures in this paper is available in the GitHub repository https://github.com/jaywan-chung/tep-chebyshev.
