# Supplementary material for: Dimension reduction of thermoelectric properties using barycentric polynomial interpolation at Chebyshev nodes
Source: Sci Rep. 2020 Aug 10;10:13456. doi: 10.1038/s41598-020-70320-7 (PMC7417583; doi:10.1038/s41598-020-70320-7)
Supplement: Supplementary file 1 — Supplementary information [file 41598_2020_70320_MOESM1_ESM.pdf]

SUPPLEMENTARY INFORMATION FOR "DIMENSION REDUCTION OF THERMOELECTRIC PROPERTIES USING BARYCENTRIC POLYNOMIAL INTERPOLATION AT CHEBYSHEV NODES"

JAYWAN CHUNG, BYUNGKI RYU, AND SUDONG PARK

1. Thermoelectric Material Properties (TEP) dataset

The TEP dataset used for the validation in the paper consists of 276 materials gathered from 263 literatures [1–263]. The TEPs were digitized using the Plot Digitizer [264].

The same dataset has been used to validate a general efficiency theory of thermoelectric energy conversion in the preprints [265, 266] and the information of the dataset is described in their Supplementary Information. But for the reader's convenience, we repeat the information on the base-material groups from the preprints.

Table S1 shows the distribution of base-material groups in the TEP dataset. Here the base-material denotes the representative material, not the exact composition. Also note that for the categorizatoin of base materials, the doping element is ignored. For examples, Bi_2Te_3, Sb_2Te_3, Bi_2Se_3 binary and their ternary alloys are categorized as Bi_2Te_3-related materials. The material doping composition is not denoted in the composition of the base material.

group of base material	# of materials	group of base material	# of materials
Bi_2Te_3	59	SnSe	8
PbTe	55	PbSe	7
skutterudite	40	half-Heusler	7
Mg_2Si	23	SiGe	6
GeTe	18	In_4Se_3	3
M_2Q	14	PbS	3
SnTe	12	oxide	2
ABQ_2	11	clathrate	2
etc.	6	**Total**	**276**

Table S1. Material groups in the TEP Dataset used in the paper. Here M = Cu, Ag or Au, Q = Te or Se, A=Group I, B=Bi or Sb.

Key words and phrases. dimension reduction, thermoelectric material property, sampling, polynomial interpolation, infinite-dimensional vectors, finite-dimensional representation.

2. Numeric values of Figure 2

Table S2 and Table S3 show not only the numeric values of Figure 2 in the paper but also sample standard deviation, and confidence intervals omitted for equidistant nodes.

# of nodes	Seebeck coefficient		Electrical resistivity		Thermal conductivity	
	95% confidence interval for population mean [%]	sample standard deviation [%]	95% confidence interval for population mean [%]	sample standard deviation [%]	95% confidence interval for population mean [%]	sample standard deviation [%]
3	2.685 ± 0.386	3.261	5.623 ± 1.148	9.685	2.503 ± 0.417	3.519
4	1.555 ± 0.230	1.942	2.905 ± 0.524	4.421	1.397 ± 0.273	2.304
5	1.211 ± 0.192	1.619	2.103 ± 0.406	3.424	1.073 ± 0.262	2.213
6	0.999 ± 0.167	1.406	1.687 ± 0.315	2.655	0.947 ± 0.193	1.633
7	0.925 ± 0.151	1.276	1.535 ± 0.292	2.467	0.873 ± 0.193	1.625
8	0.959 ± 0.185	1.562	1.475 ± 0.311	2.622	0.858 ± 0.208	1.751
9	1.018 ± 0.205	1.727	1.582 ± 0.319	2.691	0.993 ± 0.266	2.243
10	1.161 ± 0.246	2.077	1.715 ± 0.342	2.890	0.976 ± 0.203	1.712
11	1.354 ± 0.336	2.834	2.028 ± 0.494	4.168	1.300 ± 0.374	3.153
12	1.653 ± 0.382	3.223	2.206 ± 0.446	3.762	1.503 ± 0.441	3.722
13	2.307 ± 0.614	5.184	3.379 ± 1.012	8.540	1.942 ± 0.630	5.316
14	2.927 ± 0.749	6.320	3.898 ± 0.762	6.434	2.389 ± 0.814	6.865
15	4.025 ± 1.139	9.610	6.418 ± 1.858	15.680	3.633 ± 1.252	10.562
16	6.177 ± 1.852	15.632	7.211 ± 1.347	11.369	5.598 ± 2.077	17.530

Table S2. Relative L^1-norm error in the polynomial interpolation of TEPs at *equidistant* nodes, considered over 276 TEPs. Refer to the top of Figure 2 of the paper.

Table S2 shows equidistant-node interpolation work poorly for many nodes. The best result is achieved with 7 or 8 equidistant nodes. But the error becomes more severe as more nodes are used.

Table S3 shows Chebyshev-node interpolation gives less error as more nodes are used.

Since the three Chebyshev nodes are exactly the same as the three equidistant nodes, the three-node cases for Table S2 and Table S3 are the same.

# of nodes	Seebeck coefficient		Electrical resistivity		Thermal conductivity	
	95% confidence interval for population mean [%]	sample standard deviation [%]	95% confidence interval for population mean [%]	sample standard deviation [%]	95% confidence interval for population mean [%]	sample standard deviation [%]
3	2.685 ± 0.386	3.261	5.623 ± 1.148	9.685	2.503 ± 0.417	3.519
4	1.625 ± 0.232	1.954	3.143 ± 0.593	5.001	1.459 ± 0.309	2.606
5	1.179 ± 0.183	1.546	2.150 ± 0.416	3.511	1.047 ± 0.224	1.886
6	0.870 ± 0.125	1.056	1.508 ± 0.251	2.115	0.838 ± 0.157	1.328
7	0.715 ± 0.108	0.909	1.296 ± 0.238	2.007	0.734 ± 0.164	1.386
8	0.620 ± 0.100	0.847	1.075 ± 0.194	1.640	0.616 ± 0.133	1.125
9	0.531 ± 0.081	0.683	0.896 ± 0.177	1.491	0.520 ± 0.103	0.869
10	0.470 ± 0.082	0.692	0.812 ± 0.160	1.350	0.466 ± 0.096	0.808
11	0.406 ± 0.071	0.602	0.681 ± 0.134	1.132	0.391 ± 0.078	0.659
12	0.373 ± 0.072	0.605	0.598 ± 0.116	0.977	0.334 ± 0.068	0.575
13	0.324 ± 0.063	0.531	0.530 ± 0.092	0.774	0.313 ± 0.073	0.612
14	0.299 ± 0.068	0.576	0.474 ± 0.078	0.655	0.265 ± 0.053	0.448
15	0.271 ± 0.065	0.545	0.417 ± 0.069	0.579	0.264 ± 0.070	0.589
16	0.243 ± 0.064	0.540	0.384 ± 0.060	0.506	0.221 ± 0.051	0.433

TABLE S3. Relative L^1-norm error in the polynomial interpolation of TEPs at *Chebyshev* nodes, considered over 276 TEPs. Refer to Figure 2 in the paper.

3. Numeric values of Figure 3

Table S4 and Table S5 show not only the numeric values of Figure 3 in the paper but also sample standard deviation, and confidence intervals omitted for equidistant nodes.

# of nodes	Seebeck coefficient		Electrical resistivity		Thermal conductivity	
	95% confidence interval for population mean [%]	sample standard deviation [%]	95% confidence interval for population mean [%]	sample standard deviation [%]	95% confidence interval for population mean [%]	sample standard deviation [%]
3	5.116 ± 0.711	5.999	8.058 ± 1.204	10.160	5.013 ± 0.858	7.239
4	3.687 ± 0.619	5.221	5.669 ± 0.946	7.985	3.728 ± 0.783	6.606
5	3.149 ± 0.570	4.813	4.440 ± 0.747	6.302	3.111 ± 0.653	5.509
6	2.721 ± 0.534	4.504	3.889 ± 0.658	5.553	2.757 ± 0.534	4.510
7	2.875 ± 0.562	4.739	4.022 ± 0.716	6.045	2.877 ± 0.649	5.476
8	3.207 ± 0.612	5.167	4.245 ± 0.804	6.784	3.010 ± 0.664	5.603
9	4.076 ± 0.779	6.572	5.161 ± 0.960	8.102	3.856 ± 0.884	7.459
10	5.382 ± 1.074	9.065	6.899 ± 1.345	11.353	4.587 ± 0.947	7.995
11	7.451 ± 1.670	14.096	9.564 ± 2.127	17.946	7.044 ± 1.747	14.744
12	10.976 ± 2.347	19.810	12.011 ± 2.265	19.115	9.478 ± 2.492	21.028
13	17.042 ± 4.023	33.953	20.311 ± 5.547	46.814	14.357 ± 3.776	31.864
14	24.537 ± 5.920	49.956	27.148 ± 5.059	42.689	19.830 ± 6.519	55.013
15	36.376 ± 9.541	80.513	47.280 ± 12.436	104.949	33.051 ± 9.511	80.260
16	62.361 ± 17.538	148.002	62.315 ± 11.467	96.771	56.562 ± 18.536	156.428

Table S4. Relative L^∞-norm error in the polynomial interpolation of TEPs at *equidistant* nodes, considered over 276 TEPs. Refer to the top of Figure 3 in the paper.

Table S4 and Table S5 show the same tendency as for the L^1-norm case. The error values become larger than those for L^1-norm, but the error increase in the Chebyshev nodes are noticeably smaller than that in the equidistant nodes.

# of nodes	Seebeck coefficient		Electrical resistivity		Thermal conductivity	
	95% confidence interval for population mean [%]	sample standard deviation [%]	95% confidence interval for population mean [%]	sample standard deviation [%]	95% confidence interval for population mean [%]	sample standard deviation [%]
3	5.116 ± 0.711	5.999	8.058 ± 1.204	10.160	5.013 ± 0.858	7.239
4	3.728 ± 0.638	5.384	5.732 ± 0.976	8.238	3.586 ± 0.682	5.752
5	3.065 ± 0.581	4.904	4.425 ± 0.770	6.500	3.058 ± 0.707	5.964
6	2.549 ± 0.520	4.392	3.781 ± 0.689	5.811	2.478 ± 0.516	4.356
7	2.281 ± 0.511	4.310	3.287 ± 0.607	5.118	2.314 ± 0.499	4.209
8	2.057 ± 0.503	4.241	2.767 ± 0.480	4.051	2.079 ± 0.482	4.066
9	1.795 ± 0.479	4.039	2.558 ± 0.468	3.946	1.814 ± 0.413	3.484
10	1.669 ± 0.475	4.009	2.278 ± 0.411	3.467	1.614 ± 0.353	2.980
11	1.544 ± 0.469	3.955	1.982 ± 0.343	2.893	1.500 ± 0.380	3.206
12	1.396 ± 0.466	3.933	1.901 ± 0.315	2.661	1.372 ± 0.335	2.826
13	1.275 ± 0.468	3.947	1.716 ± 0.288	2.428	1.188 ± 0.241	2.034
14	1.262 ± 0.552	4.661	1.502 ± 0.247	2.088	1.209 ± 0.346	2.923
15	1.212 ± 0.621	5.238	1.461 ± 0.228	1.924	1.076 ± 0.276	2.327
16	1.128 ± 0.607	5.119	1.345 ± 0.217	1.835	0.977 ± 0.255	2.149

TABLE S5. Relative L^∞-norm error in the polynomial interpolation of TEPs at *Chebyshev* nodes, considered over 276 TEPs. Refer to Figure 3 in the paper.

4. Numeric values of Figure 4

Table S6 and Table S7 show the numeric values of Figure 4 in the paper along with sample standard deviation.

# of nodes	max. power density		max. efficiency		max. zT	
	95% confidence interval for population mean [%]	sample standard deviation [%]	95% confidence interval for population mean [%]	sample standard deviation [%]	95% confidence interval for population mean [%]	sample standard deviation [%]
8	0.476 ± 0.112	0.945	0.467 ± 0.119	1.006	1.120 ± 0.423	3.571
9	0.434 ± 0.090	0.758	0.399 ± 0.097	0.823	1.222 ± 0.721	6.083
10	0.358 ± 0.083	0.701	0.311 ± 0.077	0.649	1.347 ± 0.838	7.071
11	0.275 ± 0.070	0.592	0.244 ± 0.069	0.583	0.720 ± 0.301	2.541
12	0.203 ± 0.042	0.354	0.193 ± 0.041	0.349	0.662 ± 0.263	2.219
13	0.192 ± 0.038	0.317	0.171 ± 0.036	0.307	0.678 ± 0.279	2.355
14	0.158 ± 0.032	0.273	0.146 ± 0.032	0.271	0.457 ± 0.133	1.125
15	0.134 ± 0.033	0.275	0.133 ± 0.035	0.293	0.489 ± 0.222	1.871
16	0.144 ± 0.050	0.423	0.131 ± 0.040	0.334	0.371 ± 0.146	1.228

Table S6. Relative error in three indices of module performances under the polynomial interpolation of TEPs at *Chebyshev* nodes, considered over 276 TEPs. Refer to Figure 4 in the paper.

# of nodes	I for max. power density		I for max. efficiency		T for max. zT	
	95% confidence interval for population mean [%]	sample standard deviation [%]	95% confidence interval for population mean [%]	sample standard deviation [%]	95% confidence interval for population mean [%]	sample standard deviation [%]
8	0.327 ± 0.070	0.589	0.299 ± 0.064	0.540	1.400 ± 0.374	3.159
9	0.299 ± 0.059	0.499	0.272 ± 0.055	0.464	1.274 ± 0.382	3.227
10	0.265 ± 0.062	0.523	0.243 ± 0.059	0.497	1.270 ± 0.370	3.122
11	0.202 ± 0.043	0.364	0.188 ± 0.040	0.337	0.909 ± 0.280	2.359
12	0.151 ± 0.031	0.260	0.140 ± 0.029	0.242	1.245 ± 0.376	3.176
13	0.148 ± 0.030	0.252	0.138 ± 0.029	0.241	0.763 ± 0.259	2.184
14	0.122 ± 0.025	0.212	0.112 ± 0.024	0.199	0.847 ± 0.285	2.409
15	0.098 ± 0.020	0.166	0.094 ± 0.018	0.148	0.851 ± 0.279	2.352
16	0.107 ± 0.029	0.246	0.097 ± 0.026	0.215	0.625 ± 0.179	1.512

Table S7. Relative error in the corresponding values achieving the module performances under the polynomial interpolation of TEPs at *Chebyshev* nodes, considered over 276 TEPs. Refer to Figure 4 in the paper.

5. Numeric values of Figure 5

Table S8 (8 Chebyshev nodes), Table S9 (11 Chebyshev nodes) and Table S10 (14 Chebyshev nodes) show the numeric values of Figure 5 in the paper along with sample standard deviation.

Noise [%]	Seebeck coefficient		Electrical resistivity		Thermal conductivity	
	95% confidence interval for population mean [%]	sample standard deviation [%]	95% confidence interval for population mean [%]	sample standard deviation [%]	95% confidence interval for population mean [%]	sample standard deviation [%]
0	2.057 ± 0.503	4.241	2.767 ± 0.480	4.051	2.079 ± 0.482	4.066
1	2.441 ± 0.494	4.166	2.995 ± 0.469	3.960	2.366 ± 0.470	3.967
2	2.962 ± 0.475	4.005	3.469 ± 0.455	3.836	2.907 ± 0.457	3.861
3	3.639 ± 0.458	3.869	4.020 ± 0.437	3.687	3.627 ± 0.437	3.684
4	4.474 ± 0.466	3.929	4.682 ± 0.425	3.584	4.419 ± 0.444	3.747
5	5.298 ± 0.433	3.653	5.376 ± 0.393	3.315	5.126 ± 0.387	3.267
6	6.001 ± 0.445	3.755	6.224 ± 0.404	3.411	5.889 ± 0.389	3.281
7	6.821 ± 0.440	3.710	6.892 ± 0.385	3.246	6.798 ± 0.429	3.624
8	7.694 ± 0.437	3.685	7.526 ± 0.412	3.473	7.558 ± 0.425	3.585
9	8.703 ± 0.429	3.618	8.279 ± 0.379	3.197	8.268 ± 0.402	3.397
10	9.305 ± 0.425	3.587	9.080 ± 0.384	3.242	9.152 ± 0.414	3.497

Table S8. Effect of noise on relative L^∞-norm error in the polynomial interpolation of TEPs at *8 Chebyshev* nodes, considered over 276 TEPs. Refer to Figure 5 in the paper.

Noise [%]	Seebeck coefficient		Electrical resistivity		Thermal conductivity	
	95% confidence interval for population mean [%]	sample standard deviation [%]	95% confidence interval for population mean [%]	sample standard deviation [%]	95% confidence interval for population mean [%]	sample standard deviation [%]
0	1.544 ± 0.469	3.955	1.982 ± 0.343	2.893	1.500 ± 0.380	3.206
1	2.031 ± 0.453	3.821	2.325 ± 0.332	2.798	1.916 ± 0.374	3.159
2	2.748 ± 0.436	3.682	2.962 ± 0.304	2.563	2.617 ± 0.354	2.991
3	3.613 ± 0.437	3.689	3.646 ± 0.295	2.490	3.375 ± 0.343	2.897
4	4.451 ± 0.412	3.479	4.341 ± 0.295	2.486	4.236 ± 0.328	2.771
5	5.199 ± 0.409	3.449	5.091 ± 0.258	2.174	5.040 ± 0.323	2.727
6	6.104 ± 0.400	3.376	5.946 ± 0.295	2.492	5.997 ± 0.327	2.758
7	6.985 ± 0.400	3.377	6.851 ± 0.285	2.407	6.729 ± 0.349	2.945
8	8.021 ± 0.398	3.357	7.528 ± 0.281	2.369	7.893 ± 0.341	2.882
9	8.944 ± 0.397	3.352	8.633 ± 0.303	2.554	8.591 ± 0.315	2.662
10	9.835 ± 0.409	3.452	9.273 ± 0.291	2.459	9.327 ± 0.286	2.416

Table S9. Effect of noise on relative L^∞-norm error in the polynomial interpolation of TEPs at *11 Chebyshev* nodes, considered over 276 TEPs. Refer to Figure 5 in the paper.

Noise [%]	Seebeck coefficient		Electrical resistivity		Thermal conductivity	
	95% confidence interval for population mean [%]	sample standard deviation [%]	95% confidence interval for population mean [%]	sample standard deviation [%]	95% confidence interval for population mean [%]	sample standard deviation [%]
0	1.262 ± 0.552	4.661	1.502 ± 0.247	2.088	1.209 ± 0.346	2.923
1	1.841 ± 0.540	4.556	1.874 ± 0.228	1.921	1.700 ± 0.334	2.819
2	2.698 ± 0.527	4.447	2.533 ± 0.205	1.729	2.509 ± 0.324	2.737
3	3.678 ± 0.516	4.354	3.380 ± 0.185	1.562	3.341 ± 0.303	2.557
4	4.518 ± 0.505	4.259	4.253 ± 0.180	1.519	4.271 ± 0.306	2.586
5	5.488 ± 0.507	4.281	5.117 ± 0.181	1.527	5.112 ± 0.294	2.483
6	6.445 ± 0.498	4.199	5.930 ± 0.195	1.645	5.988 ± 0.272	2.292
7	7.282 ± 0.491	4.143	6.883 ± 0.193	1.632	7.163 ± 0.305	2.575
8	8.347 ± 0.496	4.183	7.768 ± 0.197	1.663	7.995 ± 0.309	2.607
9	9.389 ± 0.496	4.188	8.536 ± 0.219	1.848	8.682 ± 0.277	2.341
10	10.166 ± 0.490	4.139	9.518 ± 0.249	2.105	9.956 ± 0.282	2.379

TABLE S10. Effect of noise on relative L^∞-norm error in the polynomial interpolation of TEPs at *14 Chebyshev* nodes, considered over 276 TEPs. Refer to Figure 5 in the paper.

6. Numeric values of Figure 6

Table S11, S12 (8 Chebyshev nodes), Table S13, S14 (11 Chebyshev nodes) and Table S15, S16 (14 Chebyshev nodes) show the numeric values of Figure 6 in the paper along with sample standard deviation.

Noise [%]	max. power density		max. efficiency		max. zT	
	95% confidence interval for population mean [%]	sample standard deviation [%]	95% confidence interval for population mean [%]	sample standard deviation [%]	95% confidence interval for population mean [%]	sample standard deviation [%]
0	0.476 ± 0.112	0.945	0.467 ± 0.119	1.006	1.120 ± 0.423	3.571
1	0.743 ± 0.113	0.953	0.680 ± 0.118	0.999	1.915 ± 0.427	3.604
2	1.132 ± 0.119	1.005	0.991 ± 0.129	1.085	2.849 ± 0.470	3.968
3	1.557 ± 0.156	1.313	1.380 ± 0.146	1.229	3.809 ± 0.509	4.296
4	1.927 ± 0.191	1.609	1.694 ± 0.174	1.467	4.884 ± 0.508	4.288
5	2.418 ± 0.243	2.047	2.056 ± 0.235	1.980	5.858 ± 0.587	4.952
6	2.663 ± 0.239	2.014	2.304 ± 0.210	1.769	7.146 ± 0.657	5.541
7	3.303 ± 0.296	2.496	2.682 ± 0.254	2.148	8.579 ± 0.774	6.535
8	3.626 ± 0.338	2.848	3.099 ± 0.297	2.506	10.136 ± 0.860	7.261
9	4.191 ± 0.358	3.024	3.448 ± 0.312	2.636	11.236 ± 0.998	8.423
10	4.530 ± 0.430	3.627	3.846 ± 0.399	3.364	13.067 ± 1.176	9.928

Table S11. Effect of noise on relative error in three indices of module performances under the polynomial interpolation of TEPs at *8 Chebyshev* nodes, considered over 276 TEPs. Refer to Figure 6 in the paper.

Noise [%]	I for max. power density		I for max. efficiency		T for max. zT	
	95% confidence interval for population mean [%]	sample standard deviation [%]	95% confidence interval for population mean [%]	sample standard deviation [%]	95% confidence interval for population mean [%]	sample standard deviation [%]
0	0.327 ± 0.070	0.589	0.299 ± 0.064	0.540	1.400 ± 0.374	3.159
1	0.475 ± 0.071	0.603	0.423 ± 0.065	0.546	1.630 ± 0.318	2.686
2	0.732 ± 0.079	0.670	0.639 ± 0.073	0.612	2.024 ± 0.366	3.086
3	0.995 ± 0.103	0.866	0.853 ± 0.093	0.783	2.333 ± 0.428	3.611
4	1.207 ± 0.109	0.923	1.051 ± 0.096	0.809	2.980 ± 0.481	4.060
5	1.470 ± 0.154	1.302	1.269 ± 0.142	1.200	3.286 ± 0.520	4.388
6	1.721 ± 0.158	1.337	1.533 ± 0.141	1.192	3.094 ± 0.481	4.061
7	2.001 ± 0.185	1.558	1.735 ± 0.160	1.348	4.101 ± 0.544	4.589
8	2.255 ± 0.207	1.745	1.953 ± 0.183	1.543	4.453 ± 0.668	5.634
9	2.709 ± 0.237	2.001	2.389 ± 0.210	1.769	4.711 ± 0.626	5.283
10	2.825 ± 0.262	2.209	2.451 ± 0.231	1.953	4.610 ± 0.620	5.234

Table S12. Effect of noise on relative error in the corresponding values achieving the module performances under the polynomial interpolation of TEPs at *8 Chebyshev* nodes, considered over 276 TEPs. Refer to Figure 6 in the paper.

Noise [%]	max. power density		max. efficiency		max. zT	
	95% confidence interval for population mean [%]	sample standard deviation [%]	95% confidence interval for population mean [%]	sample standard deviation [%]	95% confidence interval for population mean [%]	sample standard deviation [%]
0	0.275 ± 0.070	0.592	0.244 ± 0.069	0.583	0.720 ± 0.301	2.541
1	0.521 ± 0.077	0.652	0.444 ± 0.075	0.637	1.518 ± 0.319	2.689
2	0.887 ± 0.093	0.789	0.724 ± 0.084	0.706	2.556 ± 0.337	2.848
3	1.283 ± 0.131	1.104	1.073 ± 0.116	0.977	4.029 ± 0.395	3.334
4	1.612 ± 0.142	1.203	1.371 ± 0.125	1.052	5.167 ± 0.459	3.870
5	1.790 ± 0.161	1.355	1.449 ± 0.135	1.137	6.142 ± 0.522	4.409
6	2.301 ± 0.196	1.654	1.930 ± 0.179	1.510	8.257 ± 0.658	5.553
7	2.657 ± 0.267	2.250	2.326 ± 0.234	1.971	8.871 ± 0.742	6.261
8	3.068 ± 0.286	2.412	2.617 ± 0.264	2.226	11.093 ± 0.870	7.340
9	3.344 ± 0.305	2.576	2.820 ± 0.278	2.350	12.400 ± 1.076	9.082
10	3.812 ± 0.348	2.936	3.172 ± 0.299	2.522	14.120 ± 1.167	9.847

TABLE S13. Effect of noise on relative error in three indices of module performances under the polynomial interpolation of TEPs at *11 Chebyshev* nodes, considered over 276 TEPs. Refer to Figure 6 in the paper.

Noise [%]	I for max. power density		I for max. efficiency		T for max. zT	
	95% confidence interval for population mean [%]	sample standard deviation [%]	95% confidence interval for population mean [%]	sample standard deviation [%]	95% confidence interval for population mean [%]	sample standard deviation [%]
0	0.202 ± 0.043	0.364	0.188 ± 0.040	0.337	0.909 ± 0.280	2.359
1	0.337 ± 0.047	0.396	0.305 ± 0.044	0.368	1.532 ± 0.317	2.675
2	0.565 ± 0.059	0.501	0.496 ± 0.054	0.456	1.953 ± 0.309	2.609
3	0.824 ± 0.081	0.685	0.715 ± 0.072	0.609	2.356 ± 0.401	3.386
4	1.004 ± 0.095	0.803	0.876 ± 0.085	0.719	2.694 ± 0.419	3.535
5	1.154 ± 0.112	0.945	1.011 ± 0.101	0.854	3.633 ± 0.508	4.287
6	1.475 ± 0.133	1.123	1.285 ± 0.118	0.996	3.223 ± 0.492	4.155
7	1.734 ± 0.161	1.356	1.525 ± 0.142	1.200	3.892 ± 0.562	4.741
8	1.920 ± 0.179	1.511	1.666 ± 0.158	1.332	4.138 ± 0.566	4.774
9	2.098 ± 0.185	1.562	1.865 ± 0.166	1.400	4.346 ± 0.621	5.237
10	2.371 ± 0.224	1.889	2.061 ± 0.202	1.703	4.556 ± 0.614	5.181

TABLE S14. Effect of noise on relative error in the corresponding values achieving the module performances under the polynomial interpolation of TEPs at *11 Chebyshev* nodes, considered over 276 TEPs. Refer to Figure 6 in the paper.

Noise [%]	max. power density		max. efficiency		max. zT	
	95% confidence interval for population mean [%]	sample standard deviation [%]	95% confidence interval for population mean [%]	sample standard deviation [%]	95% confidence interval for population mean [%]	sample standard deviation [%]
0	0.158 ± 0.032	0.273	0.146 ± 0.032	0.271	0.457 ± 0.133	1.125
1	0.408 ± 0.038	0.317	0.342 ± 0.036	0.307	1.331 ± 0.150	1.267
2	0.770 ± 0.065	0.552	0.623 ± 0.054	0.459	2.619 ± 0.235	1.984
3	1.093 ± 0.101	0.853	0.930 ± 0.091	0.770	3.782 ± 0.310	2.620
4	1.368 ± 0.120	1.010	1.187 ± 0.107	0.905	5.788 ± 0.633	5.346
5	1.824 ± 0.150	1.266	1.489 ± 0.131	1.107	6.898 ± 0.603	5.090
6	2.012 ± 0.193	1.626	1.736 ± 0.165	1.392	9.001 ± 0.683	5.767
7	2.163 ± 0.198	1.674	1.787 ± 0.166	1.403	9.346 ± 0.780	6.582
8	2.699 ± 0.239	2.019	2.293 ± 0.217	1.832	11.140 ± 0.838	7.069
9	3.132 ± 0.293	2.472	2.539 ± 0.233	1.970	14.332 ± 1.108	9.347
10	3.188 ± 0.266	2.246	2.597 ± 0.229	1.935	15.338 ± 1.245	10.510

TABLE S15. Effect of noise on relative error in three indices of module performances under the polynomial interpolation of TEPs at *14 Chebyshev* nodes, considered over 276 TEPs. Refer to Figure 6 in the paper.

Noise [%]	I for max. power density		I for max. efficiency		T for max. zT	
	95% confidence interval for population mean [%]	sample standard deviation [%]	95% confidence interval for population mean [%]	sample standard deviation [%]	95% confidence interval for population mean [%]	sample standard deviation [%]
0	0.122 ± 0.025	0.212	0.112 ± 0.024	0.199	0.847 ± 0.285	2.409
1	0.270 ± 0.027	0.231	0.236 ± 0.025	0.215	1.496 ± 0.276	2.332
2	0.486 ± 0.045	0.382	0.424 ± 0.041	0.343	1.898 ± 0.330	2.789
3	0.691 ± 0.062	0.527	0.604 ± 0.056	0.471	2.285 ± 0.349	2.948
4	0.895 ± 0.075	0.630	0.785 ± 0.067	0.567	2.664 ± 0.401	3.383
5	1.118 ± 0.096	0.809	0.957 ± 0.085	0.717	3.369 ± 0.500	4.216
6	1.285 ± 0.118	0.999	1.114 ± 0.104	0.877	3.566 ± 0.513	4.331
7	1.383 ± 0.129	1.088	1.219 ± 0.116	0.975	4.030 ± 0.580	4.892
8	1.663 ± 0.145	1.222	1.426 ± 0.127	1.075	4.078 ± 0.602	5.077
9	1.970 ± 0.177	1.497	1.714 ± 0.156	1.317	4.709 ± 0.612	5.167
10	2.041 ± 0.184	1.551	1.785 ± 0.164	1.383	4.360 ± 0.652	5.501

TABLE S16. Relative error in the corresponding values achieving the module performances under the polynomial interpolation of TEPs at *14 Chebyshev* nodes, considered over 276 TEPs. Refer to Figure 6 in the paper.

7. Relative L^∞-norm errors from 7-Chebyshev-node interpolation

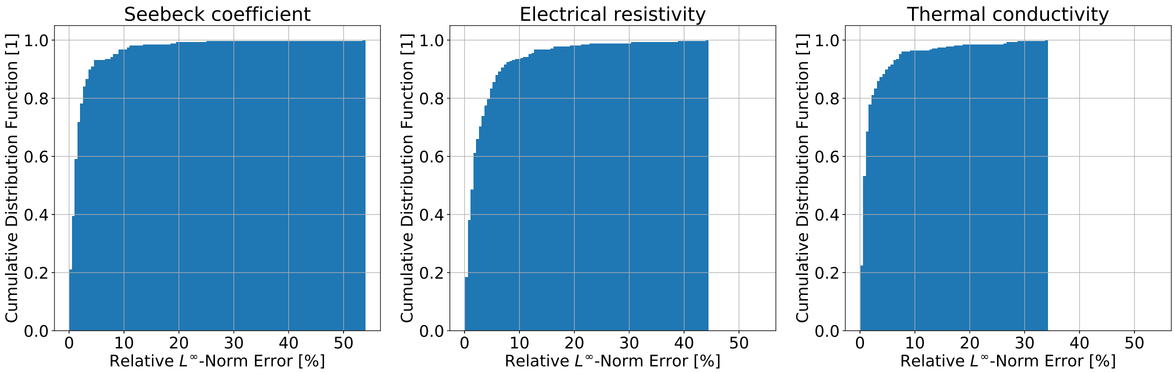

Figure S1. The proportion in the 276 sets of TEP curves that the relative L^∞-norm error from 7-Chebyshev-node interpolation is less than or equal to a given error.

percentile (q) [%]	Seebeck coefficient relative L^∞-norm error [%]	Electrical resistivity relative L^∞-norm error [%]	Thermal conductivity relative L^∞-norm error [%]
5	0.08	0.23	0.34
10	0.24	0.39	0.43
15	0.37	0.54	0.50
20	0.50	0.68	0.57
25	0.68	0.78	0.63
30	0.78	0.98	0.69
35	0.86	1.06	0.73
40	1.06	1.23	0.83
45	1.13	1.46	0.95
50	1.27	1.71	1.05
55	1.38	1.91	1.14
60	1.60	2.06	1.25
65	1.85	2.50	1.42
70	1.98	3.05	1.71
75	2.24	3.87	1.93
80	2.65	4.79	2.33
85	3.15	5.53	3.30
90	4.07	6.90	5.14
95	8.34	12.12	7.34
100	54.01	44.42	34.22

Table S17. q-th percentile of relative L^∞-norm errors when 7-Chebyshev-node interpolation is used. 45% of the interpolation results has less than 1.5% error. 80% of the interpolation results has less than 4.8% error.

Material Reference	Seebeck coefficient relative L^∞-norm error [%]	Electrical resistivity relative L^∞-norm error [%]	Thermal conductivity relative L^∞-norm error [%]
Figure 7: Bi-Sb-Te bulk alloy [17]	0.88	0.71	0.83
Figure 8(a): $Ag_2Se_{0.5}Te_{0.5}$ [141]	7.14	17.19	20.23
Figure 8(b): Cu_2Se [255]	30.33	28.63	50.94
Figure 8(c): SnSe crystal [22]	7.89	19.16	3.35

TABLE S18. Relative L^∞-norm errors for selected sets of TEPs when 7 Chebyshev-node interpolation is used. Refer to Figure 7 and Figure 8(a)–(c) in the paper.

8. Prediction of Thermoelectric property curves for p-type nanostructured bulk alloys of Bi-Sb-Te

To understand the thermoelectric transport behaviors of the Bi-Sb-Te nanostructured alloy reported in [17], we perform the charge and heat transport simulations using first-principles calculations and the Boltzmann transport equation. We simulate the electronic and phononic contributions separately.

Electronic contribution. We perform the electron Boltzmann transport calculations using the first-principles electronic structure calculations. The electronic structure is calculated within the density-functional theory (DFT) [267, 268]. We use the generalized gradient approximation parameterized by Perdew, Burke and Ernzerhof (GGA-PBE) [269], which is implemented in VASP code [270]. For the atomic potential, we use the projector augmented wave (PAW) pseudopotential [271]. To simplify the problem, we use the Bi_2Te_3 atomic structure instead of the Bi-Sb-Te alloy atomic structure. Note that the low-energy band structures and electronic thermoelectric transport properties are very similar between p-type Bi_2Te_3 and p-type Bi-Sb-Te [272, 273]. We employ the spin-orbit interaction (SOI) which is found to be very important for the low energy electronic structure of thermoelectric transport for heavy elements such as Bi, Te, and their compounds. We use the experimental lattice parameters (a=4.3835 Å, c= 30.487 Å) and the calculated atomic positions. We use the Γ-centered $36 \times 36 \times 36$ k-point mesh for the Brillouin zone integration for electron charge density.

The temperature-dependent thermoelectric properties of Bi_2Te_3 are computed within the semi-classical Boltzmann transport equation, which is implemented in BoltzTraP code [274], within the relaxation time approximation and rigid band approximation. The band gap is very important for thermoelectric transport due to the bipolar nature of narrow-gap materials. Thus, we use the experimental band gap of 0.18 eV using a scissor operator: the band dispersions are the same while the band gap is changed. For the electron relaxation time, we choose the most reliable parameter by comparing the experimental results of thermoelectric properties and the computed ones and use the following equation for the relaxation time:

$$\tau(T) = \frac{T_0}{T}\,\tau_0$$

where T_0 and τ_0 are fixed constants. By comparing the simulated transport curves and measured values, we adopt $\tau_0 = 2 \times 10^{-14}$ sec and $T_0 = 300$ K. Note that for the transport coefficient calculation, we should calculate the charge scattering time taking account of electron-phonon interaction and etc. However, due to the heavy computational cost, we do not compute the electron-phonon coupling. Here we assume the constant-energy relaxation time approximation where the electron scattering has no direct dependence on band energy. But to consider the phonon activation at high temperature, we use the relaxation time proportional to the reciprocal of temperature T. Further details are given in [273].

Since Bi_2Te_3 is a layered material, it has transport anisotropy. Since the experimental sample [17] is in polycrystalline phase, there will be weak directional behavior in transport. Thus, in our prediction model, we obtain directionally averaged thermoelectric curves: $f_{\text{av}} = \frac{2}{3} f_a + \frac{1}{3} f_c$.

The electcronic thermoelectric properties are varying with carrier concentration. The Figure 7 in the manuscript is drawn with the p-type Bi_2Te_3 with carrier concentration of $2 \times 10^{19}\,\text{cm}^{-3}$.

Phonon transport. We numerically solve the phonon Boltzmann transport equation for Bi_2Te_3 within first-principles calculations. We also use the DFT, PAW, GGA-PBE with experimental lattice parameters. The phonon lifetimes are calculated within third order anharmonic phonon Hamiltonian. We use 240-atom $4 \times 4 \times 1$ hexagonal supercells with experimental lattice parameters. Due to the computational cost, we only use the Γ point. Since force noise is too critical for third order phonon calculations, we minimize the remaining force for non-perturbed system until the force is less than 0.00002 eV/Å. Then third order force constants are obtained using the 240-atom supercells with two perturbed atomic positions. Then, a Hamiltonian is constructed by expansion upto third order. From the anharmonic part of the Hamiltonian, phonon lifetime is computed using the Fermi's Golden rule [275] for each phonon q point on the $11 \times 11 \times 11$ q-mesh. Then, phonon anharmonicity induced phonon relaxation time is obtained with three-phonon process. Since the experimental sample [17] is nanostructured, we assume that there are nanosized grains. Assuming that the effective distance between the grain boundaries is 10

nm, we compute phonon thermal conductivity using three-phonon process and the boundary scattering rate of 1/10 nm^{-1}. Finally, at 300 K, the phonon thermal conductivity is calculated to be 0.771 W/m/K along the a-direction and 0.414 W/m/K along the c-direction. Since the experimental report is for polycrystalline, we also average the phonon thermal conductivity tensor for three principal directions. The computational details can be obtained from [276].

Thermoelectric properties. Note that the thermal conductivity is a sum of electronic and lattice contributions. Using the electronic and phonon contribution on thermoelectric properties, finally we calculate the three important thermoelectric property curves for the hole carrier concentration of 2×10^{19} cm^{-3} and the effective boundary mean free path of 10 nm.

Although the predicted values are not exactly the same as the measured ones, the temperature dependence of the thermoelectric property curves is well reproduced within first-principles and Boltzmann transport calculations. See Figure 7 in the manuscript.

9. Abnormal temperature and abnormal curve detection algorithm

In this section, we describe the algorithm we used to detect the abnormal temperature points in Figure 8 (vertical lines of the figure). The abnormal temperature points are detected by two different methods: inspection of the error in the curve values or their derivatives.

Value-error detection. We compute the relative L^∞-norm errors between the (assumed) exact curve and the interpolating curve at the raw data points. The temperature points are sorted by the errors in descending order. As we are assuming the presence of an anomaly, the unknown exact curves are assumed to be picewise linear. Then the first few temperature points which have the highest errors are selected as the abnormal temperature points. If two temperature points are too close, the one with a higher error is selected and the other one is discarded. In Figure 8, we decide two temperature points T_1, T_2 are too close if $\frac{|T_1 - T_2|}{\Delta T} \leq 0.05$ where the ΔT is the length of the temperature interval under interpolation.

As one TEP consists of three curves (Seebeck coefficient, electrical resistivity, and thermal conductivity), we consider the errors from the three curves altogether as they are from one curve. In Figure 8, the abnormal temperature point with a vertical dotted line is found in this way.

If a detected abnormal temperature point gives a high error for all the three TEP curves, it is a piece of strong evidence that a phase transition occurs around that point.

Derivative-error detection. The value-error detection algorithm above detects a value-jumping point rather than a derivative-jumping point. If a raw data of the derivatives was given, derivative-jumping points could be detected just as before by considering the derivatives instead of the values. But no derivative information is available for TEPs.

As a second-best approach, we consider the peaks of the relative L^∞-norm error between the derivative of the exact curve and the derivative of the interpolating curve in the whole temperature interval. As the piecewise linear exact curve is not differentiable, the error distribution is not differentiable on temperature. To make the error distribution regular, we use Gaussian filter implemented in the `gaussian_filter1d` function [277] of the SciPy library. The standard deviation for Gaussian kernel used in Figure 8 is $0.01\Delta T$. Then the Gaussian-filtered error distribution has distinct error peaks and the highest-peak temperature points are selected as the abnormal points. As before too-close points are discarded. In Figure 8, the abnormal temperature point with a vertical dashed line is found in this way.

Abnormal curve detection. Using the 7-Chebyshev-node interpolation in the value-error detection algorithm, we found that many curves have a small error as shown in Table S17. For example, 80% of the curves has less than 4.8% error. On the other hand, some reported phase transition case, the error is significantly high as 20%; see Table S18. Hence a high-value-error curve can be considered as an abnormal curve.

Detecting a high-derivative-error curve is difficult. This is because our dataset does not provide derivative information, and no baseline for a high derivative error. Instead, if one suspects the presence of a derivative jump, one may use the derivative-error detection algorithm to spot the jumping point.

References

[1] Kanishka Biswas, Jiaqing He, Qichun Zhang, Guoyu Wang, Ctirad Uher, Vinayak P. Dravid, and Mercouri G. Kanatzidis. Strained endotaxial nanostructures with high thermoelectric figure of merit. *Nat Chem*, 3(2):160–166, February 2011.

[2] Kanishka Biswas, Jiaqing He, Ivan D. Blum, Chun-I Wu, Timothy P. Hogan, David N. Seidman, Vinayak P. Dravid, and Mercouri G. Kanatzidis. High-performance bulk thermoelectrics with all-scale hierarchical architectures. *Nature*, 489(7416):414–418, September 2012.

[3] Chenguang Fu, Shengqiang Bai, Yintu Liu, Yunshan Tang, Lidong Chen, Xinbing Zhao, and Tiejun Zhu. Realizing high figure of merit in heavy-band p-type half-Heusler thermoelectric materials. *Nat Commun*, 6:8144, September 2015.

[4] Yaniv Gelbstein, Joseph Davidow, Steven N. Girard, Duck Young Chung, and Mercouri Kanatzidis. Controlling metallurgical phase separation reactions of the $Ge_{0.87}Pb_{0.13}Te$ alloy for high thermoelectric performance. *Adv. Energy Mater.*, 3(6):815–820, June 2013.

[5] Ying He, Ping Lu, Xun Shi, Fangfang Xu, Tiansong Zhang, Gerald Jeffrey Snyder, Ctirad Uher, and Lidong Chen. Ultrahigh thermoelectric performance in mosaic crystals. *Adv. Mater.*, 27:3639–3644, May 2015.

[6] Joseph P. Heremans, Vladimir Jovovic, Eric S. Toberer, Ali Saramat, Ken Kurosaki, Anek Charoenphakdee, Shinsuke Yamanaka, and G. Jeffrey Snyder. Enhancement of thermoelectric efficiency in PbTe by distortion of the electronic density of states. *Science*, 321(5888):554–557, July 2008.

[7] Kuei Fang Hsu, Sim Loo, Fu Guo, Wei Chen, Jeffrey S. Dyck, Ctirad Uher, Tim Hogan, E. K. Polychroniadis, and Mercouri G. Kanatzidis. Cubic $AgPb_mSbTe_{2+m}$: Bulk thermoelectric materials with high figure of merit. *Science*, 303(5659):818–821, June 2004.

[8] Li-Peng Hu, Tie-Jun Zhu, Ya-Guang Wang, Han-Hui Xie, Zhao-Jun Xu, and Xin-Bing Zhao. Shifting up the optimum figure of merit of p-type bismuth telluride-based thermoelectric materials for power generation by suppressing intrinsic conduction. *NPG Asia Mater*, 6(2):e88, February 2014.

[9] Xiaokai Hu, Priyanka Jood, Michihiro Ohta, Masaru Kunii, Kazuo Nagase, Hirotaka Nishiate, Mercouri G. Kanatzidis, and Atsushi Yamamoto. Power generation from nanostructured PbTe-based thermoelectrics: comprehensive development from materials to modules. *Energy Environ. Sci.*, 9(2):517–529, February 2016.

[10] Sang Il Kim, Kyu Hyoung Lee, Hyeon A. Mun, Hyun Sik Kim, Sung Woo Hwang, Jong Wook Roh, Dae Jin Yang, Weon Ho Shin, Xiang Shu Li, Young Hee Lee, G. Jeffrey Snyder, and Sung Wng Kim. Dense dislocation arrays embedded in grain boundaries for high-performance bulk thermoelectrics. *Science*, 348(6230):109–114, March 2015.

[11] Siqi Lin, Wen Li, Zhiwei Chen, Jiawen Shen, Binghui Ge, and Yanzhong Pei. Tellurium as a high-performance elemental thermoelectric. *Nat Commun*, 7:10287, January 2016.

[12] Wei-Shu Liu, Qinyong Zhang, Yucheng Lan, Shuo Chen, Xiao Yan, Qian Zhang, Hui Wang, Dezhi Wang, Gang Chen, and Zhifeng Ren. Thermoelectric property studies on Cu-doped n-type $Cu_xBi_2Te_{2.7}Se_{0.3}$ nanocomposites. *Advanced Energy Materials*, 1(4):577–587, 2011.

[13] Wei Liu, Xiaojian Tan, Kang Yin, Huijun Liu, Xinfeng Tang, Jing Shi, Qingjie Zhang, and Ctirad Uher. Convergence of conduction bands as a means of enhancing thermoelectric performance of n-type $Mg_2Si_{1-x}Sn_x$ solid solutions. *Phys. Rev. Lett.*, 108(16):166601, April 2012.

[14] Yu Pan and Jing-Feng Li. Thermoelectric performance enhancement in n-type $Bi_2(TeSe)_3$ alloys owing to nanoscale inhomogeneity combined with a spark plasma-textured microstructure. *NPG Asia Mater*, 8(6):e275, June 2016.

[15] Yanzhong Pei, Xiaoya Shi, Aaron LaLonde, Heng Wang, Lidong Chen, and G. Jeffrey Snyder. Convergence of electronic bands for high performance bulk thermoelectrics. *Nature*, 473(7345):66–69, May 2011.

[16] Yanzhong Pei, Aaron LaLonde, Shiho Iwanaga, and G. Jeffrey Snyder. High thermoelectric figure of merit in heavy hole dominated PbTe. *Energy Environ. Sci.*, 4(6):2085–2089, June 2011.

[17] Bed Poudel, Qing Hao, Yi Ma, Yucheng Lan, Austin Minnich, Bo Yu, Xiao Yan, Dezhi Wang, Andrew Muto, Daryoosh Vashaee, Xiaoyuan Chen, Junming Liu, Mildred S. Dresselhaus, Gang Chen, and Zhifeng Ren. High-thermoelectric performance of nanostructured bismuth antimony telluride bulk alloys. *Science*, 320(5876):634–638, February 2008.

[18] Jong-Soo Rhyee, Kyu Hyoung Lee, Sang Mock Lee, Eunseog Cho, Sang Il Kim, Eunsung Lee, Yong Seung Kwon, Ji Hoon Shim, and Gabriel Kotliar. Peierls distortion as a route to high thermoelectric performance in $In_4Se_{3-\delta}$ crystals. *Nature*, 459(7249):965–968, June 2009.

[19] Hongchao Wang, Je-Hyeong Bahk, Chanyoung Kang, Junphil Hwang, Kangmin Kim, Jungwon Kim, Peter Burke, John E. Bowers, Arthur C. Gossard, Ali Shakouri, and others. Right sizes of nano-and microstructures for high-performance and rigid bulk thermoelectrics. *Proceedings of the National Academy of Sciences*, 111(30):10949–10954, July 2014.

[20] Li-Dong Zhao, Jiaqing He, Shiqiang Hao, Chun-I Wu, Timothy P. Hogan, C. Wolverton, Vinayak P. Dravid, and Mercouri G. Kanatzidis. Raising the thermoelectric performance of *p*-type PbS with endotaxial nanostructuring and valence-band offset engineering using CdS and ZnS. *J. Am. Chem. Soc.*, 134(39):16327–16336, October 2012.

[21] Li-Dong Zhao, Jiaqing He, Chun-I Wu, Timothy P. Hogan, Xiaoyuan Zhou, Ctirad Uher, Vinayak P. Dravid, and Mercouri G. Kanatzidis. Thermoelectrics with earth abundant elements: High performance *p*-type PbS nanostructured with SrS and CaS. *J. Am. Chem. Soc.*, 134(18):7902–7912, May 2012.

[22] Li-Dong Zhao, Shih-Han Lo, Yongsheng Zhang, Hui Sun, Gangjian Tan, Ctirad Uher, C. Wolverton, Vinayak P. Dravid, and Mercouri G. Kanatzidis. Ultralow thermal conductivity and high thermoelectric figure of merit in SnSe crystals. *Nature*, 508(7496):373–377, April 2014.

[23] Li-Dong Zhao, Gangjian Tan, Shiqiang Hao, Jiaqing He, Yanling Pei, Hang Chi, Heng Wang, Shengkai Gong, Huibin Xu, Vinayak P. Dravid, Ctirad Uher, G. Jeffrey Snyder, Chris Wolverton, and Mercouri G. Kanatzidis. Ultrahigh power factor and thermoelectric performance in hole-doped single-crystal SnSe. *Science*, page aad3749, November 2015.

[24] J. L. Cui, W. J. Xiu, L. D. Mao, P. Z. Ying, L. Jiang, and X. Qian. Thermoelectric properties of Ag-doped *n*-type $(Bi_2Te_3)_{0.9}$-$(Bi_{2-x}Ag_xSe_3)_{0.1}$ (x=0–0.4) alloys prepared by spark plasma sintering. *Journal of Solid State Chemistry*, 180(3):1158–1162, March 2007.

[25] J. L. Cui, H. F. Xue, W. J. Xiu, L. D. Mao, P. Z. Ying, and L. Jiang. Crystal structure analysis and thermoelectric properties of *p*-type pseudo-binary $(Al_2Te_3)_x$-$Bi_{0.5}Sb_{1.5}Te_3)_{1-x}$ (x=0–0.2) alloys prepared by spark plasma sintering. *Journal of Alloys and Compounds*, 460(1??):426–431, July 2008.

[26] A.-Young Eum, Il-Ho Kim, Soon-Mok Choi, Young Soo Lim, Won-Seon Seo, Jae-Soung Park, and Seung-Ho Yang. Transport and thermoelectric properties of $Bi_2Te_{2.7}Se_{0.3}$ prepared by mechanical alloying and hot pressing. *Journal of the Korean Physical Society*, 66(11):1726–1731, June 2015.

[27] Shufen Fan, Junnan Zhao, Jun Guo, Qingyu Yan, Jan Ma, and Huey Hoon Hng. *p*-type $Bi_{0.4}Sb_{1.6}Te_3$ nanocomposites with enhanced figure of merit. *Applied Physics Letters*, 96(18):182104, May 2010.

[28] Mi-Kyung Han, Sol Kim, Ha-Yeong Kim, and Sung-Jin Kim. An alternative strategy to construct interfaces in bulk thermoelectric material: nanostructured heterophase Bi_2Te_3/Bi_2S_3. *RSC Adv.*, 3(14):4673–4679, March 2013.

[29] HUNG CHANG HSU, JING-YI HUANG, and TSAI-KUN HUANG. Enhancing figure of merit of $Bi_{0.5}Sb_{1.5}Te_3$ through nano-composite approach. *China Steel Technical Report*, 27:57–63, 2014.

[30] Yun Zheng, Qiang Zhang, Xianli Su, Hongyao Xie, Shengcheng Shu, Tianle Chen, Gangjian Tan, Yonggao Yan, Xinfeng Tang, Ctirad Uher, and G. Jeffrey Snyder. Mechanically robust BiSbTe alloys with superior thermoelectric performance: A case study of stable hierarchical nanostructured thermoelectric materials. *Adv. Energy Mater.*, 5(5):1401391, November 2014.

[31] Lipeng Hu, Haijun Wu, Tiejun Zhu, Chenguang Fu, Jiaqing He, Pingjun Ying, and Xinbing Zhao. Tuning multiscale microstructures to enhance thermoelectric performance of *n*-type bismuth-telluride-based solid solutions. *Adv. Energy Mater.*, 5(17):1500411, June 2015.

[32] Sungwoo Hwang, Sang-Il Kim, Kyunghan Ahn, Jong Wook Roh, Dae-Jin Yang, Sang-Mock Lee, and Kyu-Hyoung Lee. Enhancing the thermoelectric properties of p-type bulk Bi-Sb-Te nanocomposites via solution-based metal nanoparticle decoration. *Journal of Elec Materi*, 42(7):1411–1416, July 2013.

[33] Jieun Ko, Jong-Young Kim, Soon-Mok Choi, Young Soo Lim, Won-Seon Seo, and Kyu Hyoung Lee. Nanograined thermoelectric $Bi_2Te_{2.7}Se_{0.3}$ with ultralow phonon transport prepared from chemically exfoliated nanoplatelets. *Journal of Materials Chemistry A*, 1(41):12791, 2013.

[34] Qihao Zhang, Xin Ai, Lianjun Wang, Yanxia Chang, Wei Luo, Wan Jiang, and Lidong Chen. Improved thermoelectric performance of silver nanoparticles-dispersed Bi_2Te_3 composites deriving from hierarchical two-phased heterostructure. *Adv. Funct. Mater.*, 25(6):966–976, February 2015.

[35] X. B. Zhao, X. H. Ji, Y. H. Zhang, T. J. Zhu, J. P. Tu, and X. B. Zhang. Bismuth telluride nanotubes and the effects on the thermoelectric properties of nanotube-containing nanocomposites. *Applied Physics Letters*, 86(6):062111, February 2005.

[36] J. K. Lee, S. D. Park, B. S. Kim, M. W. Oh, S. H. Cho, B. K. Min, H. W. Lee, and M. H. Kim. Control of thermoelectric properties through the addition of Ag in the $Bi_{0.5}Sb_{1.5}Te_3$ alloy. *Electron. Mater. Lett.*, 6(4):201–207, December 2010.

[37] Kyu-Hyoung Lee, Sungwoo Hwang, Byungki Ryu, Kyunghan Ahn, Jongwook Roh, Daejin Yang, Sang-Mock Lee, Hyunsik Kim, and Sang-Il Kim. Enhancement of the thermoelectric performance of $Bi_{0.4}Sb_{1.6}Te_3$ alloys by In and Ga doping. *Journal of Elec Materi*, 42(7):1617–1621, July 2013.

[38] Dong Sun Lee, Tae-Hoon Kim, Cheol-Hee Park, Chan-Yeup Chung, Young Soo Lim, Won-Seon Seo, and Hyung-Ho Park. Crystal structure, properties and nanostructuring of a new layered chalcogenide semiconductor, Bi_2MnTe_4. *CrystEngComm*, 15(27):5532–5538, June 2013.

[39] Xiao Yan, Bed Poudel, Yi Ma, W. S. Liu, G. Joshi, Hui Wang, Yucheng Lan, Dezhi Wang, Gang Chen, and Z. F. Ren. Experimental studies on anisotropic thermoelectric properties and structures of n-type $Bi_2Te_{2.7}Se_{0.3}$. *Nano Lett.*, 10(9):3373–3378, September 2010.

[40] Go-Eun Lee, Il-Ho Kim, Young Soo Lim, Won-Seon Seo, Byeong-Jun Choi, and Chang-Won Hwang. Preparation and thermoelectric properties of doped Bi_2Te_3-Bi_2Se_3 solid solutions. *Journal of Elec Materi*, 43(6):1650–1655, October 2013.

[41] Go-Eun Lee, Il-Ho Kim, Young Soo Lim, Won-Seon Seo, Byeong-Jun Choi, and Chang-Won Hwang. Preparation and thermoelectric properties of Bi_2Te_3-Bi_2Se_3 solid solutions. *Journal of the Korean Physical Society*, 64(10):1416–1420, May 2014.

[42] Go-Eun Lee, Il-Ho Kim, Young Soo Lim, Won-Seon Seo, Byeong-Jun Choi, and Chang-Won Hwang. Preparation and thermoelectric properties of iodine-doped Bi_2Te_3-Bi_2Se_3 solid solutions. *Journal of the Korean Physical Society*, 65(5):696–701, September 2014.

[43] Go-Eun Lee, A.-Young Eum, Kwon-Min Song, Il-Ho Kim, Young Soo Lim, Won-Seon Seo, Byeong-Jun Choi, and Chang-Won Hwang. Preparation and thermoelectric properties of n-type $Bi_2Te_{2.7}Se_{0.3}$:Dm. *Journal of Elec Materi*, 44(6):1579–1584, November 2014.

[44] Go-Eun Lee, Il-Ho Kim, Young Soo Lim, Won-Seon Seo, Byeong-Jun Choi, and Chang-Won Hwang. Thermoelectric properties of I-doped $Bi_2Te_{2.85}Se_{0.15}$ solid solutions. *Journal of the Korean Physical Society*, 64(11):1692–1696, June 2014.

[45] S. Sumithra, Nathan J. Takas, Dinesh K. Misra, Westly M. Nolting, P.f.p. Poudeu, and Kevin L. Stokes. Enhancement in thermoelectric figure of merit in nanostructured Bi_2Te_3 with semimetal nanoinclusions. *Adv. Energy Mater.*, 1(6):1141–1147, 2011.

[46] K. C. Lukas, W. S. Liu, Z. F. Ren, and C. P. Opeil. Transport properties of Ni, Co, Fe, Mn doped $Cu_{0.01}Bi_2Te_{2.7}Se_{0.3}$ for thermoelectric device applications. *Journal of Applied Physics*, 112(5):054509, September 2012.

[47] Yuho Min, Jong Wook Roh, Heeseung Yang, Minwoo Park, Sang Il Kim, Sungwoo Hwang, Sang Mock Lee, Kyu Hyoung Lee, and Unyong Jeong. Surfactant-free scalable synthesis of Bi_2Te_3 and Bi_2Se_3 nanoflakes and enhanced thermoelectric properties of their nanocomposites. *Advanced Materials*, 25(10):1425–1429, 2013.

[48] Hyeona Mun, Kyu Hyoung Lee, Suk Jun Kim, Jong-Young Kim, Jeong Hoon Lee, Jae-Hong Lim, Hee Jung Park, Jong Wook Roh, and Sung Wng Kim. Fe-doping effect on thermoelectric properties

of p-type $Bi_{0.48}Sb_{1.52}Te_3$. *Materials*, 8(3):959–965, March 2015.

[49] Sergey V. Ovsyannikov, Natalia V. Morozova, Igor V. Korobeinikov, Lidia N. Lukyanova, Andrey Y. Manakov, Anna Y. Likhacheva, Alexey I. Ancharov, Alexander P. Vokhmyanin, Ivan F. Berger, Oleg A. Usov, Vsevolod A. Kutasov, Vladimir A. Kulbachinskii, Taku Okada, and Vladimir V. Shchennikov. Enhanced power factor and high-pressure effects in $(Bi, Sb)_2(Te, Se)_3$ thermoelectrics. *Applied Physics Letters*, 106(14):143901, April 2015.

[50] Pooja Puneet, Ramakrishna Podila, Mehmet Karakaya, Song Zhu, Jian He, Terry M. Tritt, Mildred S. Dresselhaus, and Apparao M. Rao. Preferential scattering by interfacial charged defects for enhanced thermoelectric performance in few-layered n-type Bi_2Te_3. *Sci. Rep.*, 3, November 2013.

[51] Ho Sun Shin, Seong Gi Jeon, Jin Yu, Yong-Sung Kim, Hyun Min Park, and Jae Yong Song. Twin-driven thermoelectric figure-of-merit enhancement of Bi_2Te_3 nanowires. *Nanoscale*, 6(11):6158, 2014.

[52] Jae Sung Son, Moon Kee Choi, Mi-Kyung Han, Kunsu Park, Jae-Yeol Kim, Seong Joon Lim, Myunghwan Oh, Young Kuk, Chan Park, Sung-Jin Kim, and Taeghwan Hyeon. n-type nanostructured thermoelectric materials prepared from chemically synthesized ultrathin Bi_2Te_3 nanoplates. *Nano Lett.*, 12(2):640–647, February 2012.

[53] J. H. Son, M. W. Oh, B. S. Kim, S. D. Park, B. K. Min, M. H. Kim, and H. W. Lee. Effect of ball milling time on the thermoelectric properties of p-type $(Bi, Sb)_2Te_3$. *Journal of Alloys and Compounds*, 566:168–174, July 2013.

[54] Ajay Soni, Zhao Yanyuan, Yu Ligen, Michael Khor Khiam Aik, Mildred S. Dresselhaus, and Qihua Xiong. Enhanced thermoelectric properties of solution grown $Bi_2Te_{3-x}Se_x$ nanoplatelet composites. *Nano Lett.*, 12(3):1203–1209, March 2012.

[55] Yukun Xiao, Guoxin Chen, Haiming Qin, Menglei Wu, Zhepeng Xiao, Jun Jiang, Jingtao Xu, Haochuan Jiang, and Gaojie Xu. Enhanced thermoelectric figure of merit in p-type $Bi_{0.48}Sb_{1.52}Te_3$ alloy with WSe_2 addition. *J. Mater. Chem. A*, 2(22):8512–8516, April 2014.

[56] Xinfeng Tang, Wenjie Xie, Han Li, Wenyu Zhao, Qingjie Zhang, and Masayuki Niino. Preparation and thermoelectric transport properties of high-performance p-type Bi_2Te_3 with layered nanostructure. *Applied Physics Letters*, 90(1):012102, January 2007.

[57] Shanyu Wang, Han Li, Ruiming Lu, Gang Zheng, and Xinfeng Tang. Metal nanoparticle decorated n-type Bi_2Te_3-based materials with enhanced thermoelectric performances. *Nanotechnology*, 24(28):285702, July 2013.

[58] Fang Wu, Hongzhang Song, Jianfeng Jia, Feng Gao, Yingjiu Zhang, and Xing Hu. Thermoelectric properties of Ce-doped n-type $Ce_xBi_{2-x}Te_{2.7}Se_{0.3}$ nanocomposites. *Phys. Status Solidi A*, 210(6):1183–1189, June 2013.

[59] Ö. Ceyda Yelgel and G. P. Srivastava. Thermoelectric properties of n-type $Bi_2(Te_{0.85}Se_{0.15})_3$ single crystals doped with CuBr and SbI_3. *Phys. Rev. B*, 85(12):125207, March 2012.

[60] Genqiang Zhang, Benjamin Kirk, Luis A. Jauregui, Haoran Yang, Xianfan Xu, Yong P. Chen, and Yue Wu. Rational synthesis of ultrathin n-type Bi_2Te_3 nanowires with enhanced thermoelectric properties. *Nano Lett.*, 12(1):56–60, January 2012.

[61] Ping Wei, Jiong Yang, Liang Guo, Shanyu Wang, Lihua Wu, Xianfan Xu, Wenyu Zhao, Qingjie Zhang, Wenqing Zhang, Mildred S. Dresselhaus, and Jihui Yang. Minimum thermal conductivity in weak topological insulators with bismuth-based stack structure. *Adv. Funct. Mater.*, 26(29):5360–5367, May 2016.

[62] Jinle Lan, Yuan-Hua Lin, Yong Liu, Shaoliang Xu, and Ce-Wen Nan. High thermoelectric performance of nanostructured In_2O_3-based ceramics. *Journal of the American Ceramic Society*, 95(8):2465–2469, August 2012.

[63] Chao Yu, Xiaoguang Zhang, Mei Leng, Alateng Shaga, Dan Liu, Fanglin Chen, and Cheng Wang. Preparation and thermoelectric properties of inhomogeneous bismuth telluride alloyed nanorods. *Journal of Alloys and Compounds*, 570:86–93, September 2013.

[64] Atsuko Kosuga, Kazuki Nakai, Mie Matsuzawa, Yousuke Fujii, Ryoji Funahashi, Takuya Tachizawa, Yoshiki Kubota, and Kouichi Kifune. Enhanced thermoelectric performance of In-substituted $GeSb_6Te_{10}$ with homologous structure. *APL Materials*, 2(8):086102, August 2014.

[65] Marcus Scheele, Niels Oeschler, Igor Veremchuk, Sven-Ole Peters, Alexander Littig, Andreas Kornowski, Christian Klinke, and Horst Weller. Thermoelectric properties of lead chalcogenide core shell nanostructures. *ACS Nano*, 5(11):8541–8551, November 2011.

[66] Kyunghan Ahn, Changpeng Li, Ctirad Uher, and Mercouri G. Kanatzidis. Improvement in the thermoelectric figure of merit by La/Ag cosubstitution in PbTe. *Chemistry of Materials*, 21(7):1361–1367, April 2009.

[67] Kyunghan Ahn, Mi-Kyung Han, Jiaqing He, John Androulakis, Sedat Ballikaya, Ctirad Uher, Vinayak P. Dravid, and Mercouri G. Kanatzidis. Exploring resonance levels and nanostructuring in the PbTe-CdTe system and enhancement of the thermoelectric figure of merit. *J. Am. Chem. Soc.*, 132(14):5227–5235, April 2010.

[68] Kyunghan Ahn, Kanishka Biswas, Jiaqing He, In Chung, Vinayak Dravid, and Mercouri G. Kanatzidis. Enhanced thermoelectric properties of p-type nanostructured PbTe-MTe (M = Cd, Hg) materials. *Energy Environ. Sci.*, 6(5):1529–1537, April 2013.

[69] John Androulakis, Iliya Todorov, Duck-Young Chung, Sedat Ballikaya, Guoyu Wang, Ctirad Uher, and Mercouri Kanatzidis. Thermoelectric enhancement in PbTe with K or Na codoping from tuning the interaction of the light- and heavy-hole valence bands. *Phys. Rev. B*, 82(11):115209, September 2010.

[70] John Androulakis, Yeseul Lee, Iliya Todorov, Duck-Young Chung, and Mercouri Kanatzidis. High-temperature thermoelectric properties of n-type PbSe doped with Ga, In, and Pb. *Physical Review B*, 83, May 2011.

[71] Ashoka Bali, Il-Ho Kim, Peter Rogl, and Ramesh Chandra Mallik. Thermoelectric properties of two-phase PbTe with indium inclusions. *Journal of Electronic Materials*, 43:1630–1638, June 2014.

[72] Ashoka Bali, Heng Wang, G. Jeffrey Snyder, and Ramesh Chandra Mallik. Thermoelectric properties of indium doped $PbTe_{1-y}Se_y$ alloys. *Journal of Applied Physics*, 116(3):033707, July 2014.

[73] Di Wu, Li-Dong Zhao, Xiao Tong, Wei Li, Lijun Wu, Qing Tan, Yanling Pei, Li Huang, Jing-Feng Li, Yimei Zhu, Mercouri Kanatzidis, and Jiaqing He. Superior thermoelectric performance in PbTe-PbS pseudo-binary: Extremely low thermal conductivity and modulated carrier. *Energy & Environmental Science*, 8:2056, 2015.

[74] Yongkwan Dong, Michael A. McGuire, Abds-Sami Malik, and Francis J. DiSalvo. Transport properties of undoped and Br-doped PbTe sintered at high-temperature and pressure ≥ 4.0 GPa. *Journal of Solid State Chemistry*, 182(10):2602–2607, October 2009.

[75] H. S. Dow, M. W. Oh, B. S. Kim, S. D. Park, B. K. Min, H. W. Lee, and D. M. Wee. Effect of Ag or Sb addition on the thermoelectric properties of PbTe. *Journal of Applied Physics*, 108(11):113709, December 2010.

[76] Oliver Falkenbach, David Hartung, Peter J. Klar, Guenter Koch, and Sabine Schlecht. Thermoelectric properties of nanostructured bismuth-doped lead telluride $Bi_x(PbTe)_{1-x}$ prepared by co-ball-milling. *Journal of Electronic Materials*, 43(6):1674–1680, November 2014.

[77] Haotian Fan, Taichao Su, Hongtao Li, Baoli Du, Bingguo Liu, Hairui Sun, Yuewen Zhang, Liang Li, Shangsheng Li, Meihua Hu, Hongan Ma, and Xiaopeng Jia. Enhanced thermoelectric performance of PbSe co-doped with Ag and Sb. *Journal of Alloys and Compounds*, 639:106–110, August 2015.

[78] Haiyu Fang, Tianli Feng, Haoran Yang, Xiulin Ruan, and Yue Wu. Synthesis and thermoelectric properties of compositional-modulated lead telluride bismuth telluride nanowire heterostructures. *Nano Letters*, 13(5):2058–2063, May 2013.

[79] Christopher M. Jaworski, Michele D. Nielsen, Hsin Wang, Steven N. Girard, Wei Cai, Wally D. Porter, Mercouri G. Kanatzidis, and Joseph P. Heremans. Valence-band structure of highly efficient p-type thermoelectric PbTe-PbS alloys. *Phys. Rev. B*, 87(4):045203, January 2013.

[80] Zhengzhong Jian, Zhiwei Chen, Wen Li, Jiong Yang, Wenqing Zhang, and Yanzhong Pei. Significant band engineering effect of YbTe for high performance thermoelectric PbTe. *J. Mater. Chem. C*, 3(48):12410–12417, December 2015.

[81] T. Keiber, F. Bridges, B.C. Sales, and H. Wang. Complex role for thallium in PbTe:Tl from local probe studies. *Physical Review B*, 87, Apr 2013.

[82] Min-Seok Kim, Woo-Jin Lee, Ki-Hyun Cho, Jae-Pyoung Ahn, and Yun-Mo Sung. Spinodally decomposed PbSe-PbTe nanoparticles for high-performance thermoelectrics: Enhanced phonon scattering and unusual transport behavior. *ACS Nano*, 10(7):7197–7207, July 2016.
[83] J. K. Lee, M. W. Oh, S. D. Park, B. S. Kim, B. K. Min, M. H. Kim, and H. W. Lee. Improvement of thermoelectric properties through controlling the carrier concentration of $AgPb_{18}SbTe_{20}$ alloys by Sb addition. *Electron. Mater. Lett.*, 8(6):659–663, December 2012.
[84] Yeseul Lee, Shih-Han Lo, Changqiang Chen, Hui Sun, Duck-Young Chung, Thomas C. Chasapis, Ctirad Uher, Vinayak P. Dravid, and Mercouri G. Kanatzidis. Contrasting role of antimony and bismuth dopants on the thermoelectric performance of lead selenide. *Nat Commun*, 5:3640, May 2014.
[85] X.X. Li, J.Q. Li, F.S. Liu, W.Q. Ao, H.T. Li, and L.C. Pan. Enhanced thermoelectric properties of $(PbTe)_{0.88}(PbS)_{0.12}$ composites by Bi doping. *Journal of Alloys and Compounds*, 547:86–90, January 2013.
[86] Zong-Yue Li, Jing-Feng Li, Wen-Yang Zhao, Qing Tan, Tian-Ran Wei, Chao-Feng Wu, and Zhi-Bo Xing. PbTe-based thermoelectric nanocomposites with reduced thermal conductivity by SiC nanodispersion. *Applied Physics Letters*, 104(11):113905, March 2014.
[87] Jing Liu, Xiaoguang Wang, and Liangming Peng. Effect of annealing on thermoelectric properties of eutectic $PbTeSb_2Te_3$ composite with self-assembled lamellar structure. *Intermetallics*, 41:63–69, October 2013.
[88] Shih-Han Lo, Jiaqing He, Kanishka Biswas, Mercouri G. Kanatzidis, and Vinayak P. Dravid. Phonon scattering and thermal conductivity in p-type nanostructured PbTe-BaTe Bulk thermoelectric materials. *Adv. Funct. Mater.*, 22(24):5175–5184, December 2012.
[89] Peng-Xian Lu, Ling-Bo Qu, and Qiao-Huan Cheng. Enhancement of thermoelectric figure of merit in binary-phased $La_{0.3}Ce_{0.37}Fe_3CoS_{12}$-PbTe materials. *Journal of Alloys and Compounds*, 558:50–55, May 2013.
[90] Yanzhong Pei, Nicholas A. Heinz, Aaron LaLonde, and G. Jeffrey Snyder. Combination of large nanostructures and complex band structure for high performance thermoelectric lead telluride. *Energy Environ. Sci.*, 4(9):3640–3645, August 2011.
[91] Yanzhong Pei, Jessica Lensch-Falk, Eric S. Toberer, Douglas L. Medlin, and G. Jeffrey Snyder. High thermoelectric performance in PbTe due to large nanoscale Ag_2Te precipitates and La doping. *Advanced Functional Materials*, 21(2):241–249, 2011.
[92] Yanzhong Pei, Andrew F. May, and G. Jeffrey Snyder. Self-tuning the carrier concentration of $PbTe/Ag_2Te$ composites with excess Ag for high thermoelectric performance. *Advanced Energy Materials*, 1(2):291–296, March 2011.
[93] Yanzhong Pei, Aaron D. LaLonde, Nicholas A. Heinz, Xiaoya Shi, Shiho Iwanaga, Heng Wang, Lidong Chen, and G. Jeffrey Snyder. Stabilizing the optimal carrier concentration for high thermoelectric efficiency. *Adv. Mater.*, 23(47):5674–5678, December 2011.
[94] Yanzhong Pei, Aaron D. LaLonde, Heng Wang, and G. Jeffrey Snyder. Low effective mass leading to high thermoelectric performance. *Energy Environ. Sci.*, 5(7):7963–7969, June 2012.
[95] Yanzhong Pei, Heng Wang, Zachary M. Gibbs, Aaron D. LaLonde, and G. Jeffrey Snyder. Thermopower enhancement in $Pb_{1-x}Mn_xTe$ alloys and its effect on thermoelectric efficiency. *NPG Asia Mater*, 4(9):e28, September 2012.
[96] Yanzhong Pei, Zachary M. Gibbs, Andrei Gloskovskii, Benjamin Balke, Wolfgang G. Zeier, and G. Jeffrey Snyder. Optimum carrier concentration in n-type PbTe thermoelectrics. *Adv. Energy Mater.*, 4(13):1400486, September 2014.
[97] Pierre F. P. Poudeu, Jonathan D'Angelo, Adam D. Downey, Jarrod L. Short, Timothy P. Hogan, and Mercouri G. Kanatzidis. High thermoelectric figure of merit and nanostructuring in bulk p-type $Na_{1-x}Pb_mSb_yTe_{m+2}$. *Angewandte Chemie International Edition*, 45(23):3835–3839, June 2006.
[98] P K Rawat, B Paul, and P Banerji. Thermoelectric properties of $PbSe_{0.5}Te_{0.5}$:$x(PbI_2)$ with endotaxial nanostructures: a promising n-type thermoelectric material. *Nanotechnology*, 24(21):215401, May 2013.

[99] Hongchao Wang, Je-Hyeong Bahk, Chanyoung Kang, Junphil Hwang, Kangmin Kim, Ali Shakouri, and Woochul Kim. Large enhancement in the thermoelectric properties of $Pb_{0.98}Na_{0.02}Te$ by optimizing the synthesis conditions. *Journal of Materials Chemistry A*, 1(37):11269, August 2013.

[100] Heng Wang, Zachary M. Gibbs, Yoshiki Takagiwa, and G. Jeffrey Snyder. Tuning bands of PbSe for better thermoelectric efficiency. *Energy Environ. Sci.*, 7(2):804–811, January 2014.

[101] H. J. Wu, L.-D. Zhao, F. S. Zheng, D. Wu, Y. L. Pei, X. Tong, M. G. Kanatzidis, and J. Q. He. Broad temperature plateau for thermoelectric figure of merit $ZT > 2$ in phase-separated $PbTe_{0.7}S_{0.3}$. *Nat Commun*, 5:4515, July 2014.

[102] Haijun Wu, Jesús Carrete, Zhiyun Zhang, Yongquan Qu, Xuetao Shen, Zhao Wang, Li-Dong Zhao, and Jiaqing He. Strong enhancement of phonon scattering through nanoscale grains in lead sulfide thermoelectrics. *NPG Asia Mater*, 6(6):e108, June 2014.

[103] Sima Aminorroaya Yamini, David R. G. Mitchell, Zachary M. Gibbs, Rafael Santos, Vaughan Patterson, Sean Li, Yan Zhong Pei, Shi Xue Dou, and G. Jeffrey Snyder. Heterogeneous distribution of sodium for high thermoelectric performance of p-type multiphase lead-chalcogenides. *Adv. Energy Mater.*, 5(21):1501047, August 2015.

[104] Haoran Yang, Je-Hyeong Bahk, Tristan W Day, Amr Mohammed, G. Jeffrey Snyder, Ali Shakouri, and Yue Wu. Enhanced thermoelectric properties in bulk nanowire heterostructure-based nanocomposites through minority carrier blocking. *Nano Lett.*, 15:1349, 2015.

[105] Mona Zebarjadi, Giri Joshi, Gaohua Zhu, Bo Yu, Austin Minnich, Yucheng Lan, Xiaowei Wang, Mildred Dresselhaus, Zhifeng Ren, and Gang Chen. Power factor enhancement by modulation doping in bulk nanocomposites. *Nano Lett.*, 11(6):2225–2230, June 2011.

[106] Qinyong Zhang, Hui Wang, Weishu Liu, Hengzhi Wang, Bo Yu, Qian Zhang, Zhiting Tian, George Ni, Sangyeop Lee, Keivan Esfarjani, Gang Chen, and Zhifeng Ren. Enhancement of thermoelectric figure-of-merit by resonant states of aluminium doping in lead selenide. *Energy & Environmental Science*, 5(1):5246, 2012.

[107] Qian Zhang, Feng Cao, Weishu Liu, Kevin Lukas, Bo Yu, Shuo Chen, Cyril Opeil, David Broido, Gang Chen, and Zhifeng Ren. Heavy doping and band engineering by potassium to improve the thermoelectric figure of merit in p-type PbTe, PbSe, and $PbTe_{1-y}Se_y$. *J. Am. Chem. Soc.*, 134(24):10031–10038, June 2012.

[108] Qinyong Zhang, Siqi Yang, Qian Zhang, Shuo Chen, Weishu Liu, Hui Wang, Zhiting Tian, David Broido, Gang Chen, and Zhifeng Ren. Effect of aluminum on the thermoelectric properties of nanostructured PbTe. *Nanotechnology*, 24(34):345705, August 2013.

[109] Qian Zhang, Eyob Kebede Chere, Kenneth McEnaney, Mengliang Yao, Feng Cao, Yizhou Ni, Shuo Chen, Cyril Opeil, Gang Chen, and Zhifeng Ren. Enhancement of thermoelectric performance of n-type PbSe by Cr doping with optimized carrier concentration. *Adv. Energy Mater.*, 5(8):1401977, 2015.

[110] Rabih Al Rahal Al Orabi, Nicolas A. Mecholsky, Junphil Hwang, Woochul Kim, Jong-Soo Rhyee, Daehyun Wee, and Marco Fornari. Band degeneracy, low thermal conductivity, and high thermoelectric figure of merit in SnTe-CaTe alloys. *Chem. Mater.*, 28(1):376–384, December 2015.

[111] Ananya Banik, U. Sandhya Shenoy, Shashwat Anand, Umesh V. Waghmare, and Kanishka Biswas. Mg alloying in SnTe facilitates valence band convergence and optimizes thermoelectric properties. *Chem. Mater.*, 27(2):581–587, January 2015.

[112] Ananya Banik, U. Sandhya Shenoy, Sujoy Saha, Umesh V. Waghmare, and Kanishka Biswas. High power factor and enhanced thermoelectric performance of SnTe-$AgInTe_2$: Synergistic effect of resonance level and valence band convergence. *Journal of the American Chemical Society*, 138(39):13068–13075, September 2016.

[113] Ananya Banik and Kanishka Biswas. AgI alloying in SnTe boosts the thermoelectric performance via simultaneous valence band convergence and carrier concentration optimization. *Journal of Solid State Chemistry*, 242:43–49, 2016.

[114] Cheng-Lung Chen, Heng Wang, Yang-Yuan Chen, Tristan Day, and Jeffery Snyder. Thermoelectric properties of p-type polycrystalline SnSe doped with Ag. *J. Mater. Chem. A*, 2(29):11171–11176, May 2014.

[115] Yue-Xing Chen, Zhen-Hua Ge, Meijie Yin, Dan Feng, Xue-Qin Huang, Wenyu Zhao, and Jiaqing He. Understanding of the extremely low thermal conductivity in high-performance polycrystalline SnSe through potassium doping. *Adv. Funct. Mater.*, 26(37):6836–6845, August 2016.

[116] Hua-Qian Leng, Min Zhou, Jie Zhao, Ye-Mao Han, and Lai-Feng Li. The thermoelectric performance of anisotropic SnSe doped with Na. *RSC Adv.*, 6(11):9112–9116, January 2016.

[117] Yanzhong Pei, Linglang Zheng, Wen Li, Siqi Lin, Zhiwei Chen, Yanying Wang, Xiangfan Xu, Hulei Yu, Yue Chen, and Binghui Ge. Interstitial point defect scattering contributing to high thermoelectric performance in SnTe. *Adv. Electron. Mater.*, 2(6):1600019, May 2016.

[118] Gangjian Tan, Li-Dong Zhao, Fengyuan Shi, Jeff W. Doak, Shih-Han Lo, Hui Sun, Chris Wolverton, Vinayak P. Dravid, Ctirad Uher, and Mercouri G. Kanatzidis. High thermoelectric performance of p-type SnTe via a synergistic band engineering and nanostructuring approach. *J. Am. Chem. Soc.*, 136(19):7006–7017, May 2014.

[119] Gangjian Tan, Fengyuan Shi, Shiqiang Hao, Hang Chi, Li-Dong Zhao, Ctirad Uher, Chris Wolverton, Vinayak P. Dravid, and Mercouri G. Kanatzidis. Codoping in SnTe: Enhancement of thermoelectric performance through synergy of resonance levels and band convergence. *J. Am. Chem. Soc.*, 137(15):5100–5112, April 2015.

[120] Gangjian Tan, Fengyuan Shi, Shiqiang Hao, Hang Chi, Trevor P. Bailey, Li-Dong Zhao, Ctirad Uher, Chris Wolverton, Vinayak P. Dravid, and Mercouri G. Kanatzidis. Valence band modification and high thermoelectric performance in SnTe heavily alloyed with MnTe. *J. Am. Chem. Soc.*, 137(35):11507–11516, September 2015.

[121] Guodong Tang, Wei Wei, Jian Zhang, Yusheng Li, Xiang Wang, Guizhou Xu, Cheng Chang, Zhihe Wang, Youwei Du, and Li-Dong Zhao. Realizing high figure of merit in phase-separated polycrystalline $Sn_{1-x}Pb_xSe$. *Journal of the American Chemical Society*, 138(41):13647–13654, September 2016.

[122] Xinke WANG, Kai GUO, Igor Veremchuk, Ulrich Burkhardt, Xianjuan FENG, Juri Grin, and Jingtai ZHAO. Thermoelectric properties of Eu- and Na-substituted SnTe. *Journal of Rare Earths*, 33(11):1175–1181, November 2015.

[123] Q. Zhang, B. Liao, Y. Lan, K. Lukas, W. Liu, K. Esfarjani, C. Opeil, D. Broido, G. Chen, and Z. Ren. High thermoelectric performance by resonant dopant indium in nanostructured SnTe. *Proceedings of the National Academy of Sciences*, 110(33):13261–13266, August 2013.

[124] Min Zhou, Zachary M. Gibbs, Heng Wang, Yemao Han, Caini Xin, Laifeng Li, and G. Jeffrey Snyder. Optimization of thermoelectric efficiency in SnTe: the case for the light band. *Phys. Chem. Chem. Phys.*, 16(38):20741–20748, August 2014.

[125] Xinhong Guan, Pengfei Lu, Liyuan Wu, Lihong Han, Gang Liu, Yuxin Song, and Shumin Wang. Thermoelectric properties of SnSe compound. *Journal of Alloys and Compounds*, 643:116–120, September 2015.

[126] Yasumitsu Suzuki and Hisao Nakamura. A supercell approach to the doping effect on the thermoelectric properties of SnSe. *Phys. Chem. Chem. Phys.*, 17(44):29647–29654, November 2015.

[127] Felix Fahrnbauer, Daniel Souchay, Gerald Wagner, and Oliver Oeckler. High thermoelectric figure of merit values of germanium antimony tellurides with kinetically stable cobalt germanide precipitates. *J. Am. Chem. Soc.*, 137(39):12633–12638, September 2015.

[128] Y. Gelbstein, Z. Dashevsky, and M.P. Dariel. In-doped $Pb_{0.5}Sn_{0.5}Te$ p-type samples prepared by powder metallurgical processing for thermoelectric applications. *Physica B: Condensed Matter*, 396(1-2):16–21, June 2007.

[129] Y. Gelbstein, Z. Dashevsky, and M.P. Dariel. Powder metallurgical processing of functionally graded p-$Pb_{1-x}Sn_xTe$ materials for thermoelectric applications. *Physica B: Condensed Matter*, 391(2):256–265, April 2007.

[130] Yaniv Gelbstein, Yoav Rosenberg, Yatir Sadia, and Moshe P. Dariel. Thermoelectric properties evolution of spark plasma sintered $(Ge_{0.6}Pb_{0.3}Sn_{0.1})Te$ following a spinodal decomposition. *The Journal of Physical Chemistry C*, 114(30):13126–13131, August 2010.

[131] Eden Hazan, Naor Madar, Maya Parag, Vladimir Casian, Ohad Ben-Yehuda, and Yaniv Gelbstein. Effective electronic mechanisms for optimizing the thermoelectric properties of GeTe-rich alloys.

Adv. Electron. Mater., 1(11):1500228, November 2015.

[132] Bogusław Kusz, Tadeusz Miruszewski, Beata Bochentyn, Marcin Łapiński, and Jakub Karczewski. Structure and thermoelectric properties of Te-Ag-Ge-Sb (TAGS) materials obtained by reduction of melted oxide substrates. *Journal of Elec Materi*, 45(2):1085–1093, February 2016.

[133] J. K. Lee, M. W. Oh, B. S. Kim, B. K. Min, H. W. Lee, and S. D. Park. Influence of Mn on crystal structure and thermoelectric properties of GeTe compounds. *Electron. Mater. Lett.*, 10(4):813–817, July 2014.

[134] Thorsten Schröder, Tobias Rosenthal, Nadja Giesbrecht, Markus Nentwig, Stefan Maier, Heng Wang, G Jeffrey Snyder, and Oliver Oeckler. Nanostructures in Te/Sb/Ge/Ag (TAGS) thermoelectric materials induced by phase transitions associated with vacancy ordering. *Inorg. Chem.*, 53(14):7722–7729, July 2014.

[135] Thorsten Schröder, Tobias Rosenthal, Nadja Giesbrecht, Stefan Maier, Ernst-Wilhelm Scheidt, Wolfgang Scherer, G Jeffrey Snyder, Wolfgang Schnick, and Oliver Oeckler. TAGS-related indium compounds and their thermoelectric properties–the solid solution series $(GeTe)_xAgIn_ySb_{1-y}Te_2$ (x = 1–2; y = 0.5 and 1). *J. Mater. Chem. A*, 2(18):6384–6395, 2014.

[136] Jared B. Williams, Edgar Lara-Curzio, Ercan Cakmak, Thomas Watkins, and Donald T. Morelli. Enhanced thermoelectric performance driven by high-temperature phase transition in the phase change material Ge_4SbTe_5. *Journal of Materials Research*, FirstView:1–6, 2015.

[137] Di Wu, Li-Dong Zhao, Shiqiang Hao, Qike Jiang, Fengshan Zheng, Jeff W. Doak, Haijun Wu, Hang Chi, Yaniv Gelbstein, Ctirad Uher, C. Wolverton, Mercouri G. Kanatzidis, and Jiaqing He. Origin of the high performance in GeTe-based thermoelectric materials upon Bi_2Te_3 doping. *J. Am. Chem. Soc.*, 136(32):11412–11419, July 2014.

[138] Yusufu Aikebaier, Ken Kurosaki, Hiroaki Muta, and Shinsuke Yamanaka. Effect of (Pb,Ge)Te addition on the phase stability and the thermoelectric properties of $AgSbTe_2$. *MRS Online Proceedings Library*, 1267:1267–DD04–11, 2010.

[139] Y. Chen, B. He, T. J. Zhu, and X. B. Zhao. Thermoelectric properties of non-stoichiometric $AgSbTe_2$ based alloys with a small amount of GeTe addition. *J. Phys. D: Appl. Phys.*, 45(11):115302, March 2012.

[140] H. S. Dow, M. W. Oh, S. D. Park, B. S. Kim, B. K. Min, H. W. Lee, and D. M. Wee. Thermoelectric properties of $AgPb_mSbTe_{m+2}$ ($12 \leq m \leq 26$) at elevated temperature. *Journal of Applied Physics*, 105(11):113703, June 2009.

[141] Fivos Drymiotis, Tristan W. Day, David R. Brown, Nicholas A. Heinz, and G. Jeffrey Snyder. Enhanced thermoelectric performance in the very low thermal conductivity $Ag_2Se_{0.5}Te_{0.5}$. *Applied Physics Letters*, 103(14):143906, October 2013.

[142] B. Du, H. Li, and X. Tang. Effect of Ce substitution for Sb on the thermoelectric properties of $AgSbTe_2$ compound. *Journal of Electronic Materials*, 43(6):2384–2389, June 2014.

[143] Satya Narayan Guin and Kanishka Biswas. Sb deficiencies control hole transport and boost the thermoelectric performance of p-type $AgSbSe_2$. *J. Mater. Chem. C*, 3(40):10415–10421, June 2015.

[144] Mi-Kyung Han, John Androulakis, Sung-Jin Kim, and Mercouri G. Kanatzidis. Lead-free thermoelectrics: High figure of merit in p-type $AgSn_mSbTe_{m+2}$. *Adv. Energy Mater.*, 2(1):157–161, January 2012.

[145] Zhang He, Luo Jun, Zhu Hang-Tian, Liu Quan-Lin, Liang Jing-Kui, Li Jing-Bo, and Liu Guang-Yao. Synthesis and thermoelectric properties of Mn-doped $AgSbTe_2$ compounds. *Chinese Phys. B*, 21(10):106101, October 2012.

[146] A. J. Hong, L. Li, H. X. Zhu, X. H. Zhou, Q. Y. He, W. S. Liu, Z. B. Yan, J. M. Liu, and Z. F. Ren. Anomalous transport and thermoelectric performances of CuAgSe compounds. *Solid State Ionics*, 261:21–25, August 2014.

[147] Xiaocun Liu, Dou Jin, and Xin Liang. Enhanced thermoelectric performance of n-type transformable $AgBiSe_2$ polymorphs by indium doping. *Applied Physics Letters*, 109(13):133901, September 2016.

[148] Rajeshkumar Mohanraman, Raman Sankar, Fang-Cheng Chou, Chih-Hao Lee, Yoshiyuki Iizuka, I. Panneer Muthuselvam, and Yang-Yuan Chen. Influence of nanoscale Ag_2Te precipitates on the

thermoelectric properties of the Sn doped P-type $AgSbTe_2$ compound. *APL Materials*, 2(9):096114, September 2014.

[149] Yanzhong Pei, Nicholas A. Heinz, and G. Jeffrey Snyder. Alloying to increase the band gap for improving thermoelectric properties of Ag_2Te. *J. Mater. Chem.*, 21(45):18256–18260, November 2011.

[150] Heng Wang, Jing-Feng Li, Minmin Zou, and Tao Sui. Synthesis and transport property of $AgSbTe_2$ as a promising thermoelectric compound. *Applied Physics Letters*, 93(20):202106, November 2008.

[151] Hsin-jay Wu, Tian-Wey Lan, Sinn-wen Chen, Yang-Yuan Chen, Tristan Day, and G. Jeffery Snyder. State of the art $Ag_{50-x}Sb_xSe_{50-y}Te_y$ alloys: Their high zT values, microstructures and related phase equilibria. *Acta Materialia*, 93:38–45, July 2015.

[152] S. N. Zhang, T. J. Zhu, S. H. Yang, C. Yu, and X. B. Zhao. Improved thermoelectric properties of $AgSbTe_2$ based compounds with nanoscale Ag_2Te in situ precipitates. *Journal of Alloys and Compounds*, 499(2):215–220, June 2010.

[153] Tatsuhiko Aizawa, Renbo Song, and Atsushi Yamamoto. Solid state synthesis of ternary thermoelectric magnesium alloy. *Materials Transactions*, 47(4):1058–1065, 2006.

[154] Masayasu Akasaka, Tsutomu Iida, Keishi Nishio, and Yoshifumi Takanashi. Composition dependent thermoelectric properties of sintered $Mg_2Si_{1-x}Ge_x$ (x=0 to 1) initiated from a melt-grown polycrystalline source. *Thin Solid Films*, 515(22):8237–8241, August 2007.

[155] Masayasu Akasaka, Tsutomu Iida, Takashi Nemoto, Junichi Soga, Junichi Sato, Kenichiro Makino, Masataka Fukano, and Yoshifumi Takanashi. Non-wetting crystal growth of Mg_2Si by vertical Bridgman method and thermoelectric characteristics. *Journal of Crystal Growth*, 304(1):196–201, June 2007.

[156] X. Cheng, N. Farahi, and H. Kleinke. Mg_2Si-based materials for the thermoelectric energy conversion. *JOM*, 68(10):2680–2687, August 2016.

[157] Xingkai Duan, Konggang Hu, Jing Kuang, Yuezhen Jiang, and Dengliang Yi. Effects of Ag-doping on thermoelectric properties of $Ca_{(2-x)}Ag_xSi$ alloys. *Journal of Electronic Materials*, 46(5):2986–2989, November 2016.

[158] T. Kajikawa, K. Shida, K. Shiraishi, T. Ito, M. Omori, and T. Hirai. Thermoelectric figure of merit of impurity doped and hot-pressed magnesium silicide elements. In *Seventeenth International Conference on Thermoelectrics. Proceedings ICT98 (Cat. No.98TH8365)*, pages 362–369, May 1998.

[159] Weishu Liu, Hee Seok Kim, Shuo Chen, Qing Jie, Bing Lv, Mengliang Yao, Zhensong Ren, Cyril P. Opeil, Stephen Wilson, Ching-Wu Chu, and Zhifeng Ren. n-type thermoelectric material $Mg_2Sn_{0.75}Ge_{0.25}$ for high power generation. *PNAS*, 112(11):3269–3274, March 2015.

[160] Weijun Luo, Meijun Yang, Fei Chen, Qiang Shen, Hongyi Jiang, and Lianmeng Zhang. Fabrication and thermoelectric properties of $Mg_2Si_{1-x}Sn_x$ solid solutions by solid state reaction and spark plasma sintering. *Materials Science and Engineering: B*, 157(1-3):96–100, February 2009.

[161] K. Mars, H. Ihou-Mouko, G. Pont, J. Tobola, and H. Scherrer. Thermoelectric properties and electronic structure of Bi- and Ag-doped $Mg_2Si_{1-x}Ge_x$ compounds. *Journal of Electronic Materials*, 38(7):1360–1364, July 2009.

[162] Yasutoshi Noda, Hiroyuki Kon, Yoshitaka Furukawa, Isao A. Nishida, and Katashi Masumoto. Temperature dependence of thermoelectric properties of $Mg_2Si_{0.6}Ge_{0.4}$. *Mater. Trans., JIM*, 33(9):851–855, March 1992.

[163] Jun-ichi Tani and Hiroyasu Kido. Thermoelectric properties of Bi-doped Mg_2Si semiconductors. *Physica B: Condensed Matter*, 364(1-4):218–224, July 2005.

[164] Jun-ichi Tani and Hiroyasu Kido. Thermoelectric properties of P-doped Mg_2Si semicouctors. *Japanese Journal of Applied Physics*, 46:3309–3314, June 2007.

[165] Jun-ichi Tani and Hiroyasu Kido. Thermoelectric properties of Sb-doped Mg_2Si semiconductors. *Intermetallics*, 15(9):1202–1207, September 2007.

[166] Mei Jun Yang, Wei Jun Luo, Qiang Shen, Hong Yi Jiang, and Lian Meng Zhang. Preparation and thermoelectric properties of Bi-doped Mg_2Si nanocomposites. *Advanced Materials Research*, 66:17–20, April 2009.

[167] Kang Yin, Xianli Su, Yonggao Yan, Yonghui You, Qiang Zhang, Ctirad Uher, Mercouri G. Kanatzidis, and Xinfeng Tang. Optimization of the electronic band structure and the lattice thermal conductivity of solid solutions according to simple calculations: a canonical example of the $Mg_2Si_{1-x-y}Ge_xSn_y$ ternary solid solution. *Chemistry of Materials*, 28(15):5538–5548, July 2016.

[168] Q. Zhang, J. He, T. J. Zhu, S. N. Zhang, X. B. Zhao, and T. M. Tritt. High figures of merit and natural nanostructures in $Mg_2Si_{0.4}Sn_{0.6}$ based thermoelectric materials. *Applied Physics Letters*, 93(10):102109, 2008.

[169] Q Zhang, J He, X B Zhao, S N Zhang, T J Zhu, H Yin, and T M Tritt. *In situ* synthesis and thermoelectric properties of La-doped $Mg_2(Si, Sn)$ composites. *Journal of Physics D: Applied Physics*, 41(18):185103, September 2008.

[170] Libin Zhang, Penghao Xiao, Li Shi, Graeme Henkelman, John B. Goodenough, and Jianshi Zhou. Suppressing the bipolar contribution to the thermoelectric properties of $Mg_2Si_{0.4}Sn_{0.6}$ by Ge substitution. *Journal of Applied Physics*, 117(15):155103, April 2015.

[171] X. B. Zhao, S. H. Yang, Y. Q. Cao, J. L. Mi, Q. Zhang, and T. J. Zhu. Synthesis of nanocomposites with improved thermoelectric properties. *Journal of Electronic Materials*, 38(7):1017–1024, July 2009.

[172] Giri Joshi, Hohyun Lee, Yucheng Lan, Xiaowei Wang, Gaohua Zhu, Dezhi Wang, Ryan W. Gould, Diana C. Cuff, Ming Y. Tang, Mildred S. Dresselhaus, Gang Chen, and Zhifeng Ren. Enhanced thermoelectric figure-of-merit in nanostructured *p*-type silicon germanium bulk alloys. *Nano Lett.*, 8(12):4670–4674, December 2008.

[173] Jinyao Tang, Hung-Ta Wang, Dong Hyun Lee, Melissa Fardy, Ziyang Huo, Thomas P. Russell, and Peidong Yang. Holey silicon as an efficient thermoelectric material. *Nano Letters*, 10(10):4279–4283, October 2010.

[174] X. W. Wang, H. Lee, Y. C. Lan, G. H. Zhu, G. Joshi, D. Z. Wang, J. Yang, A. J. Muto, M. Y. Tang, J. Klatsky, S. Song, M. S. Dresselhaus, G. Chen, and Z. F. Ren. Enhanced thermoelectric figure of merit in nanostructured *n*-type silicon germanium bulk alloy. *Applied Physics Letters*, 93(19):193121, 2008.

[175] Kyunghan Ahn, Eunseog Cho, Jong-Soo Rhyee, Sang Il Kim, Sungwoo Hwang, Hyun-Sik Kim, Sang Mock Lee, and Kyu Hyoung Lee. Improvement in the thermoelectric performance of the crystals of halogen-substituted $In_4Se_{3-x}H_{0.03}$ (H = F, Cl, Br, I): Effect of halogen-substitution on the thermoelectric properties in In_4Se_3. *J. Mater. Chem.*, 22(12):5730–5736, February 2012.

[176] Ranu Bhatt, Miral Patel, Shovit Bhattacharya, Ranita Basu, Sajid Ahmad, Pramod Bhatt, A. K. Chauhan, M. Navneethan, Y. Hayakawa, Ajay Singh, D. K. Aswal, and S. K. Gupta. Thermoelectric performance of layered Sr_xTiSe_2 above 300 K. *J. Phys.: Condens. Matter*, 26(44):445002, November 2014.

[177] Chenguang Fu, Tiejun Zhu, Yintu Liu, Hanhui Xie, and Xinbing Zhao. Band engineering of high performance *p*-type FeNbSb based half-Heusler thermoelectric materials for figure of merit $zT > 1$. *Energy & Environmental Science*, 8(1):216–220, 2015.

[178] D. Kraemer, J. Sui, K. McEnaney, H. Zhao, Q. Jie, Z. F. Ren, and G. Chen. High thermoelectric conversion efficiency of MgAgSb-based material with hot-pressed contacts. *Energy Environ. Sci.*, 8(4):1299–1308, April 2015.

[179] J. Krez, B. Balke, S. Ouardi, S. Selle, T. Höche, C. Felser, W. Hermes, and M. Schwind. Long-term stability of phase-separated half-Heusler compounds. *Phys. Chem. Chem. Phys.*, 17(44):29854–29858, November 2015.

[180] Wei-Shu Liu, Bo-Ping Zhang, Jing-Feng Li, and Li-Dong Zhao. Thermoelectric property of fine-grained $CoSb_3$ skutterudite compound fabricated by mechanical alloying and spark plasma sintering. *J. Phys. D: Appl. Phys.*, 40(2):566, January 2007.

[181] Ya Mudryk, P. Rogl, C. Paul, S. Berger, E. Bauer, G. Hilscher, C. Godart, and H. Noël. Thermoelectricity of clathrate I Si and Ge phases. *J. Phys.: Condens. Matter*, 14(34):7991, 2002.

[182] X. Shi, H. Kong, C.-P. Li, C. Uher, J. Yang, J. R. Salvador, H. Wang, L. Chen, and W. Zhang. Low thermal conductivity and high thermoelectric figure of merit in n-type $Ba_xYb_yCo_4Sb_{12}$ double-filled skutterudites. *Applied Physics Letters*, 92(18):182101, May 2008.

[183] S.Q. Bai, Y.Z. Pei, L.D. Chen, W.Q. Zhang, X.Y. Zhao, and J. Yang. Enhanced thermoelectric performance of dual-element-filled skutterudites $Ba_xCe_yCo_4Sb_{12}$. *Acta Materialia*, 57(11):3135–3139, June 2009.

[184] Siqian Bao, Junyou Yang, Wen Zhu, Xi'an Fan, and Xingkai Duan. Effect of processing parameters on formation and thermoelectric properties of $La_{0.4}FeCo_3Sb_{12}$ skutterudite by MA-HP method. *Journal of Alloys and Compounds*, 476(1-2):802–806, May 2009.

[185] M. Chitroub, F. Besse, and H. Scherrer. Thermoelectric properties of semi-conducting compound $CoSb_3$ doped with Pd and Te. *Journal of Alloys and Compounds*, 467(1-2):31–34, January 2009.

[186] N. Dong, X. Jia, T.C. Su, F.R. Yu, Y.J. Tian, Y.P. Jiang, L. Deng, and H.A. Ma. HPHT synthesis and thermoelectric properties of $CoSb_3$ and $Fe_{0.6}Co_{3.4}Sb_{12}$ skutterudites. *Journal of Alloys and Compounds*, 480(2):882–884, July 2009.

[187] Bo Duan, Pengcheng Zhai, Lisheng Liu, Qingjie Zhang, and Xuefeng Ruan. Synthesis and high temperature transport properties of Te-doped skutterudite compounds. *Journal of Materials Science: Materials in Electronics*, 23(10):1817–1822, October 2012.

[188] Jeffrey S. Dyck, Wei Chen, Ctirad Uher, Lidong Chen, Xinfeng Tang, and Toshio Hirai. Thermoelectric properties of the n-type filled skutterudite $Ba_{0.3}Co_4Sb_{12}$ doped with Ni. *Journal of Applied Physics*, 91(6):3698–3705, March 2002.

[189] Zeming He, Christian Stiewe, Dieter Platzek, Gabriele Karpinski, Eckhard Müller, Shanghua Li, Muhammet Toprak, and Mamoun Muhammed. Thermoelectric properties of hot-pressed skutterudite $CoSb_3$. *Journal of Applied Physics*, 101(5):053713, March 2007.

[190] Qinyu He, Shejun Hu, Xingui Tang, Yucheng Lan, Jian Yang, Xiaowei Wang, Zhifeng Ren, Qing Hao, and Gang Chen. The great improvement effect of pores on ZT in $Co_{1-x}Ni_xSb_3$ system. *Applied Physics Letters*, 93(4):042108, July 2008.

[191] F Laufek, J Navratil, J Plášil, T Plecháček, and Č Drašar. Synthesis, crystal structure and transport properties of skutterudite-related $CoSn_{1.5}Se_{1.5}$. *Journal of Alloys and Compounds*, 479(1-2):102–106, June 2009.

[192] X. Y. Li, L. D. Chen, J. F. Fan, W. B. Zhang, T. Kawahara, and T. Hirai. Thermoelectric properties of Te-doped $CoSb_3$ by spark plasma sintering. *Journal of Applied Physics*, 98(8):083702, October 2005.

[193] Tao Liang, Xianli Su, Yonggao Yan, Gang Zheng, Qiang Zhang, Hang Chi, Xinfeng Tang, and Ctirad Uher. Ultra-fast synthesis and thermoelectric properties of Te doped skutterudites. *J. Mater. Chem. A*, 2(42):17914–17918, October 2014.

[194] Wei-Shu Liu, Bo-Ping Zhang, Jing-Feng Li, Hai-Long Zhang, and Li-Dong Zhao. Enhanced thermoelectric properties in $CoSb_{3-x}Te_x$ alloys prepared by mechanical alloying and spark plasma sintering. *Journal of Applied Physics*, 102(10):103717, November 2007.

[195] Ramesh Chandra Mallik. Transport properties of Sn-filled and Te-doped $CoSb_3$ skutterudites. *Metals and Materials International*, 14(5):615–620, October 2008.

[196] R. C. Mallik, R. Anbalagan, G. Rogl, E. Royanian, P. Heinrich, E. Bauer, P. Rogl, and S. Suwas. Thermoelectric properties of $Fe_{0.2}Co_{3.8}Sb_{12-x}Te_x$ skutterudites. *Acta Materialia*, 61(18):6698–6711, October 2013.

[197] J L Mi, X B Zhao, T J Zhu, and J P Tu. Thermoelectric properties of $Yb_{0.15}Co_4Sb_{12}$ based nanocomposites with $CoSb_3$ nano-inclusion. *Journal of Physics D: Applied Physics*, 41(20):205403, October 2008.

[198] Y.Z. Pei, S.Q. Bai, X.Y. Zhao, W. Zhang, and L.D. Chen. Thermoelectric properties of $Eu_yCo_4Sb_{12}$ filled skutterudites. *Solid State Sciences*, 10(10):1422–1428, October 2008.

[199] P. F. Qiu, J. Yang, R. H. Liu, X. Shi, X. Y. Huang, G. J. Snyder, W. Zhang, and L. D. Chen. High-temperature electrical and thermal transport properties of fully filled skutterudites RFe_4Sb_{12} (R = Ca, Sr, Ba, La, Ce, Pr, Nd, Eu, and Yb). *Journal of Applied Physics*, 109(6):063713, March 2011.

[200] G. Rogl, A. Grytsiv, E. Bauer, P. Rogl, and M. Zehetbauer. Thermoelectric properties of novel skutterudites with didymium: $DD_y(Fe_{1-x}Co_x)_4Sb_{12}$ and $DD_y(Fe_{1-x}Ni_x)_4Sb_{12}$. *Intermetallics*, 18(1):57–64, January 2010.

[201] G. Rogl, A. Grytsiv, P. Rogl, E. Bauer, and M. Zehetbauer. A new generation of p-type didymium skutterudites with high ZT. *Intermetallics*, 19(4):546–555, April 2011.

[202] G. Rogl, A. Grytsiv, P. Rogl, N. Peranio, E. Bauer, M. Zehetbauer, and O. Eibl. n-type skutterudites $(R,Ba,Yb)_yCo_4Sb_{12}$ (R = Sr, La, Mm, DD, SrMm, SrDD) approaching $ZT \approx 2.0$. *Acta Materialia*, 63:30–43, January 2014.

[203] G. Rogl, A. Grytsiv, P. Heinrich, E. Bauer, P. Kumar, N. Peranio, O. Eibl, J. Horky, M. Zehetbauer, and P. Rogl. New bulk p-type skutterudites $DD_{0.7}Fe_{2.7}Co_{1.3}Sb_{12-x}X_x$ (X = Ge, Sn) reaching $ZT > 1.3$. *Acta Materialia*, 91:227–238, June 2015.

[204] B. C. Sales, D. Mandrus, and R. K. Williams. Filled skutterudite antimonides: A new class of thermoelectric materials. *Science*, 272(5266):1325–1328, May 1996.

[205] Xun Shi, Jiong Yang, James R. Salvador, Miaofang Chi, Jung Y. Cho, Hsin Wang, Shengqiang Bai, Jihui Yang, Wenqing Zhang, and Lidong Chen. Multiple-filled skutterudites: High thermoelectric figure of merit through separately optimizing electrical and thermal transports. *Journal of the American Chemical Society*, 133(20):7837–7846, May 2011.

[206] Christian Stiewe, Luca Bertini, Muhammet Toprak, Mogens Christensen, Dieter Platzek, Simon Williams, Carlo Gatti, Eckhard Müller, Bo B. Iversen, Mamoun Muhammed, and Michael Rowe. Nanostructured $Co_{1-x}Ni_x(Sb_{1-y}Te_y)_3$ skutterudites: Theoretical modeling, synthesis and thermoelectric properties. *Journal of Applied Physics*, 97(4):044317, February 2005.

[207] Xianli Su, Han Li, Guoyu Wang, Hang Chi, Xiaoyuan Zhou, Xinfeng Tang, Qingjie Zhang, and Ctirad Uher. Structure and transport properties of double-doped $CoSb_{2.75}Ge_{0.25-x}Te_x$ ($x = 0.125$–0.20) with in situ nanostructure. *Chem. Mater.*, 23(11):2948–2955, June 2011.

[208] X. F. Tang, L. D. Chen, T. Goto, T. Hirai, and R. Z. Yuan. Synthesis and thermoelectric properties of filled skutterudite compounds $Ce_yFe_xCo_{4-x}Sb_{12}$ by solid state reaction. *Journal of Materials Science*, 36(22):5435–5439, November 2001.

[209] Chenglong Xu, Bo Duan, Shijie Ding, Pengcheng Zhai, and Qingjie Zhang. Thermoelectric properties of skutterudites $Co_{4-x}Ni_xSb_{11.9-y}Te_ySe_{0.1}$. *Journal of Electronic Materials*, 43(6):2224–2228, June 2014.

[210] K. Yang, H. Cheng, H.H. Hng, J. Ma, J.L. Mi, X.B. Zhao, T.J. Zhu, and Y.B. Zhang. Synthesis and thermoelectric properties of double-filled skutterudites $Ce_yYb_{0.5-y}Fe_{1.5}Co_{2.5}Sb_{12}$. *Journal of Alloys and Compounds*, 467(1-2):528–532, January 2009.

[211] Jianjun Zhang, Bo Xu, Li-Min Wang, Dongli Yu, Jianqing Yang, Fengrong Yu, Zhongyuan Liu, Julong He, Bin Wen, and Yongjun Tian. High-pressure synthesis of phonon-glass electron-crystal featured thermoelectric $Li_xCo_4Sb_{12}$. *Acta Materialia*, 60(3):1246–1251, February 2012.

[212] Wenyu Zhao, Ping Wei, Qingjie Zhang, Chunlei Dong, Lisheng Liu, and Xinfeng Tang. Enhanced thermoelectric performance in barium and indium double-filled skutterudite bulk materials via orbital hybridization induced by indium filler. *Journal of the American Chemical Society*, 131(10):3713–3720, March 2009.

[213] Lina Zhou, Pengfei Qiu, Ctirad Uher, Xun Shi, and Lidong Chen. Thermoelectric properties of p-type $Yb_xLa_yFe_{2.7}Co_{1.3}Sb_{12}$ double-filled skutterudites. *Intermetallics*, 32:209–213, January 2013.

[214] Ashoka Bali, Raju Chetty, Amit Sharma, Gerda Rogl, Patrick Heinrich, Satyam Suwas, Dinesh Kumar Misra, Peter Rogl, Ernst Bauer, and Ramesh Chandra Mallik. Thermoelectric properties of In and I doped PbTe. *Journal of Applied Physics*, 120(17):175101, November 2016.

[215] Guangchao Ding, Jianxiao Si, Shidan Yang, GuWei Wang, and Haifei Wu. High thermoelectric properties of n-type Cd-doped PbTe prepared by melt spinning. *Scripta Materialia*, 122:1–4, September 2016.

[216] Seungki Jo, Sung Hoon Park, Hyeong Woo Ban, Da Hwi Gu, Bong-Seo Kim, Ji Hee Son, Hyo-Ki Hong, Zonghoon Lee, Hyoung-Su Han, Wook Jo, Ji Eun Lee, and Jae Sung Son. Simultaneous improvement in electrical and thermal properties of interface-engineered BiSbTe nanostructured thermoelectric materials. *Journal of Alloys and Compounds*, 689:899–907, December 2016.

[217] Sung-Jae Joo, Ji-Hee Son, Bok-Ki Min, Ji-Eun Lee, Bong-Seo Kim, Byungki Ryu, Su-Dong Park, and Hee-Woong Lee. Thermoelectric properties of $Bi_2Te_{2.7}Se_{0.3}$ nanocomposites embedded with MgO nanoparticles. *Journal of the Korean Physical Society*, 69(8):1314–1320, November 2016.

[218] Y. Y. Li, X. Y. Qin, D. Li, J. Zhang, C. Li, Y. F. Liu, C. J. Song, H. X. Xin, and H. F. Guo. Enhanced thermoelectric performance of $Cu_2Se/Bi_{0.4}Sb_{1.6}Te_3$ nanocomposites at elevated temperatures. *Appl. Phys. Lett.*, 108(6):062104, February 2016.

[219] Yuanyue Li, Guoxia Liu, Xiaoying Qin, and Fukai Shan. Inhibition of minority transport for elevating the thermoelectric figure of merit of CuO/BiSbTe nanocomposites at high temperatures. *RSC Adv.*, 6(113):112050–112056, November 2016.

[220] Wei Liu, Xinfeng Tang, Han Li, Kang Yin, Jeff Sharp, Xiaoyuan Zhou, and Ctirad Uher. Enhanced thermoelectric properties of n-type $Mg_{2.16}(Si_{0.4}Sn_{0.6})_{1-y}Sb_y$ due to nano-sized Sn-rich precipitates and an optimized electron concentration. *Journal of Materials Chemistry*, 22(27):13653, 2012.

[221] Y. M. Zhou, H. J. Wu, Y. L. Pei, C. Chang, Y. Xiao, X. Zhang, S. K. Gong, J. Q. He, and L. D. Zhao. Strategy to optimize the overall thermoelectric properties of SnTe via compositing with its property-counter $CuInTe_2$. *Acta Materialia*, 125:542–549, February 2017.

[222] Chongjian Zhou, Zhongqi Shi, Bangzhi Ge, Ke Wang, Danli Zhang, Guiwu Liu, and Guanjun Qiao. Scalable solution-based synthesis of component-controllable ultrathin $PbTe_{1-x}Se_x$ nanowires with high n-type thermoelectric performance. *J. Mater. Chem. A*, 5(6):2876–2884, February 2017.

[223] Jiawei Zhang, Lirong Song, Steffen Hindborg Pedersen, Hao Yin, Le Thanh Hung, and Bo Brummerstedt Iversen. Discovery of high-performance low-cost n-type Mg_3Sb_2-based thermoelectric materials with multi-valley conduction bands. *Nature Communications*, 8:13901, January 2017.

[224] Biao Xu, Matthias T. Agne, Tianli Feng, Thomas C. Chasapis, Xiulin Ruan, Yilong Zhou, Haimei Zheng, Je-Hyeong Bahk, Mercouri G. Kanatzidis, Gerald Jeffrey Snyder, and Yue Wu. Nanocomposites from solution-synthesized PbTe-BiSbTe nanoheterostructure with unity figure of merit at low-medium temperatures (500-600 K). *Advanced Materials*, 29(10):1605140, January 2017.

[225] Dewen Xie, Jingtao Xu, Zhu Liu, Guoqiang Liu, Hezhu Shao, Xiaojian Tan, Haochuan Jiang, and Jun Jiang. Stabilization of thermoelectric properties of the $Cu/Bi_{0.48}Sb_{1.52}Te_3$ composite for advantageous power generation. *Journal of Electronic Materials*, 46(5):2746–2751, September 2017.

[226] Shanyu Wang, Yongxing Sun, Jiong Yang, Bo Duan, Lihua Wu, Wenqing Zhang, and Jihui Yang. High thermoelectric performance in Te-free $(Bi,Sb)_2Se_3$ via structural transition induced band convergence and chemical bond softening. *Energy Environ. Sci.*, 9(11):3436–3447, September 2016.

[227] Sungho Seo, Youngkeun Jeong, Min-Wook Oh, and Bongyoung Yoo. Effect of hydrogen annealing of ball-milled $Bi_{0.5}Sb_{1.5}Te_3$ powders on thermoelectric properties. *Journal of Alloys and Compounds*, 706:576–583, June 2017.

[228] YanLing Pei, Cheng Chang, Zhe Wang, Meijie Yin, Minghui Wu, Gangjian Tan, Haijun Wu, YueXing Chen, Lei Zheng, Shengkai Gong, Tiejun Zhu, Xinbing Zhao, Li Huang, Jiaqing He, Mercouri G. Kanatzidis, and Li-Dong Zhao. Multiple converged conduction bands in $K_2Bi_8Se_{13}$: A promising thermoelectric material with extremely low thermal conductivity. *J. Am. Chem. Soc.*, 138(50):16364–16371, November 2016.

[229] Kunsu Park, Kyunghan Ahn, Joonil Cha, Sanghwa Lee, Sue In Chae, Sung-Pyo Cho, Siheon Ryee, Jino Im, Jaeki Lee, Su-Dong Park, Myung Joon Han, In Chung, and Taeghwan Hyeon. Extraordinary off-stoichiometric bismuth telluride for enhanced n-type thermoelectric power factor. *J. Am. Chem. Soc.*, 138(43):14458–14468, October 2016.

[230] Seung Pil Moon, Yeon Sik Ahn, Tae Wan Kim, Soon-Mok Choi, Hee Jung Park, Sung Wng Kim, and Kyu Hyoung Lee. Tunable thermoelectric transport properties of $Cu_{0.008}Bi_2Te_{2.7}Se_{0.3}$ via control of the spark plasma sintering conditions. *Journal of the Korean Physical Society*, 69(5):811–815, September 2016.

[231] T. J. Zhu, Y. Q. Cao, F. Yan, and X. B. Zhao. Nanostructuring and thermoelectric properties of semiconductor tellurides. In *Thermoelectrics, 2007. ICT 2007. 26th International Conference on*, pages 8–11. IEEE, 2007.

[232] Jae-Shik Choi, Hee-Jeong Kim, Hang-Chong Kim, Tae-Sung Oh, Dow-Bin Hyun, and Hee-Woong Lee. Thermoelectric properties of n-type $(Pb_{1-x}Ge_x)Te$ fabricated by hot pressing method. In *XVI International Conference on Thermoelectrics, 1997. Proceedings ICT '97*, pages 228–231, August 1997.

[233] Shinsuke Yamanaka, Atsuko Kosuga, and Ken Kurosaki. Thermoelectric properties of Tl_9BiTe_6. *Journal of Alloys and Compounds*, 352(1-2):275–278, March 2003.

[234] S H Yang, T J Zhu, T Sun, J He, S N Zhang, and X B Zhao. Nanostructures in high-performance $(GeTe)_x(AgSbTe_2)_{100-x}$ thermoelectric materials. *Nanotechnology*, 19(24):245707, June 2008.

[235] S. H. Yang, T. J. Zhu, S. N. Zhang, J. J. Shen, and X. B. Zhao. Natural microstructure and thermoelectric performance of $(GeTe)_{80}(Ag_ySb_{2-y}Te_{3-y})_{20}$. *Journal of Electronic Materials*, 39(9):2127–2131, September 2010.

[236] S. N. Zhang, J. He, X. H. Ji, Z. Su, S. H. Yang, T. J. Zhu, X. B. Zhao, and Terry M. Tritt. Effects of ball-milling atmosphere on the thermoelectric properties of TAGS-85 compounds. *Journal of Electronic Materials*, 38(7):1142–1147, July 2009.

[237] Min Zhou, Jing-Feng Li, and Takuji Kita. Nanostructured $AgPb_mSbTe_{m+2}$ system bulk materials with enhanced thermoelectric performance. *Journal of the American Chemical Society*, 130(13):4527–4532, April 2008.

[238] J. W. Sharp. Some properties of GeTe-based thermoelectric alloys. In *Thermoelectrics, 2003 Twenty-Second International Conference on-ICT*, pages 267–270. IEEE, 2003.

[239] James R. Salvador, J. Yang, X. Shi, H. Wang, and A.A. Wereszczak. Transport and mechanical property evaluation of $(AgSbTe)_{1-x}(GeTe)_x$ (x=0.80, 0.82, 0.85, 0.87, 0.90). *Journal of Solid State Chemistry*, 182(8):2088–2095, August 2009.

[240] E. M. Levin, B. A. Cook, J. L. Harringa, S. L. Bud'ko, R. Venkatasubramanian, and K. Schmidt-Rohr. Analysis of Ce- and Yb-Doped TAGS-85 materials with enhanced thermoelectric figure of merit. *Advanced Functional Materials*, 21(3):441–447, February 2011.

[241] Shinsuke Yamanaka, Hirokazu Kobayashi, and Ken Kurosaki. Thermoelectric properties of layered rare earth copper oxides. *Journal of alloys and compounds*, 349(1):321–324, 2003.

[242] Li-Dong Zhao, Bo-Ping Zhang, Jing-Feng Li, Min Zhou, Wei-Shu Liu, and Jing Liu. Thermoelectric and mechanical properties of nano-SiC-dispersed Bi_2Te_3 fabricated by mechanical alloying and spark plasma sintering. *Journal of Alloys and Compounds*, 455:259–264, May 2008.

[243] X. Y. Zhao, X. Shi, L. D. Chen, W. Q. Zhang, W. B. Zhang, and Y. Z. Pei. Synthesis and thermoelectric properties of Sr-filled skutterudite $Sr_yCo_4Sb_{12}$. *Journal of Applied Physics*, 99(5):053711, March 2006.

[244] Cui Yu, Tie-Jun Zhu, Rui-Zhi Shi, Yun Zhang, Xin-Bing Zhao, and Jian He. High-performance half-Heusler thermoelectric materials $Hf_{1-x}Zr_xNiSn_{1-y}Sb_y$ prepared by levitation melting and spark plasma sintering. *Acta Materialia*, 57(9):2757–2764, May 2009.

[245] Zhen Xiong, Xihong Chen, Xiangyang Huang, Shengqiang Bai, and Lidong Chen. High thermoelectric performance of $Yb_{0.26}Co_4Sb_{12}/y$GaSb nanocomposites originating from scattering electrons of low energy. *Acta Materialia*, 58(11):3995–4002, June 2010.

[246] Eric S. Toberer, Catherine A. Cox, Shawna R. Brown, Teruyuki Ikeda, Andrew F. May, Susan M. Kauzlarich, and G. Jeffrey Snyder. Traversing the metal-insulator transition in a Zintl phase: Rational enhancement of thermoelectric efficiency in $Yb_{14}Mn_{1-x}Al_xSb_{11}$. *Advanced Functional Materials*, 18(18):2795–2800, September 2008.

[247] Duck-Young Chung, Tim Hogan, Paul Brazis, Melissa Rocci-Lane, Carl Kannewurf, Marina Bastea, Ctirad Uher, and Mercouri G. Kanatzidis. $CsBi_4Te_6$: A high-performance thermoelectric material for low-temperature applications. *Science*, 287(5455):1024–1027, February 2000.

[248] Xinfeng Tang, Peng Li, Shukang Deng, and Qingjie Zhang. High temperature thermoelectric transport properties of double-atom-filled clathrate compounds $Yb_xBa_{8-x}Ga_{16}Ge_{30}$. *Journal of Applied Physics*, 104(1):013706, July 2008.

[249] J. L. Mi, X. B. Zhao, T. J. Zhu, and J. P. Tu. Improved thermoelectric figure of merit in n-type $CoSb_3$ based nanocomposites. *Applied Physics Letters*, 91(17):172116, October 2007.

[250] Wei-Shu Liu, Bo-Ping Zhang, Li-Dong Zhao, and Jing-Feng Li. Improvement of thermoelectric performance of $CoSb_{3-x}Te_x$ skutterudite compounds by additional substitution of IVB-group elements for Sb. *Chemistry of Materials*, 20(24):7526–7531, December 2008.

[251] Han Li, Xinfeng Tang, Xianli Su, and Qingjie Zhang. Preparation and thermoelectric properties of high-performance Sb additional $Yb_{0.2}Co_4Sb_{12+y}$ bulk materials with nanostructure. *Applied Physics Letters*, 92(20):202114, May 2008.

[252] L. D. Chen, X. Y. Huang, M. Zhou, X. Shi, and W. B. Zhang. The high temperature thermoelectric performances of $Zr_{0.5}Hf_{0.5}Ni_{0.8}Pd_{0.2}Sn_{0.99}Sb_{0.01}$ alloy with nanophase inclusions. *Journal of Applied Physics*, 99(6):064305, March 2006.

[253] Bin Zhong, Yong Zhang, Weiqian Li, Zhenrui Chen, Jingying Cui, Wei Li, Yuandong Xie, Qing Hao, and Qinyu He. High superionic conduction arising from aligned large lamellae and large figure of merit in bulk $Cu_{1.94}Al_{0.02}Se$. *Applied Physics Letters*, 105(12):123902, September 2014.

[254] Bo Yu, Weishu Liu, Shuo Chen, Hui Wang, Hengzhi Wang, Gang Chen, and Zhifeng Ren. Thermoelectric properties of copper selenide with ordered selenium layer and disordered copper layer. *Nano Energy*, 1(3):472–478, May 2012.

[255] Huili Liu, Xun Yuan, Ping Lu, Xun Shi, Fangfang Xu, Ying He, Yunshan Tang, Shengqiang Bai, Wenqing Zhang, Lidong Chen, Yue Lin, Lei Shi, He Lin, Xingyu Gao, Xingmin Zhang, Hang Chi, and Ctirad Uher. Ultrahigh thermoelectric performance by electron and phonon critical scattering in $Cu_2Se_{1-x}I_x$. *Advanced Materials*, 25(45):6607–6612, December 2013.

[256] Huili Liu, Xun Shi, Fangfang Xu, Linlin Zhang, Wenqing Zhang, Lidong Chen, Qiang Li, Ctirad Uher, Tristan Day, and G. Jeffrey Snyder. Copper ion liquid-like thermoelectrics. *Nature Materials*, 11(5):422–425, March 2012.

[257] Ying He, Tiansong Zhang, Xun Shi, Su-Huai Wei, and Lidong Chen. High thermoelectric performance in copper telluride. *NPG Asia Mater*, 7(8):e210, August 2015.

[258] Bhasker Gahtori, Sivaiah Bathula, Kriti Tyagi, M. Jayasimhadri, A. K. Srivastava, Sukhvir Singh, R. C. Budhani, and Ajay Dhar. Giant enhancement in thermoelectric performance of copper selenide by incorporation of different nanoscale dimensional defect features. *Nano Energy*, 13:36–46, April 2015.

[259] Tristan W. Day, Kasper A. Borup, Tiansong Zhang, Fivos Drymiotis, David R. Brown, Xun Shi, Lidong Chen, Bo B. Iversen, and G. Jeffrey Snyder. High-temperature thermoelectric properties of $Cu_{1.97}Ag_{0.03}Se_{1+y}$. *Materials for Renewable and Sustainable Energy*, 3(2):26, June 2014.

[260] Sedat Ballikaya, Hang Chi, James R. Salvador, and Ctirad Uher. Thermoelectric properties of Ag-doped Cu_2Se and Cu_2Te. *J. Mater. Chem. A*, 1(40):12478–12484, September 2013.

[261] Trevor P. Bailey, Si Hui, Hongyao Xie, Alan Olvera, Pierre F. P. Poudeu, Xinfeng Tang, and Ctirad Uher. Enhanced ZT and attempts to chemically stabilize Cu_2Se via Sn doping. *J. Mater. Chem. A*, 4(43):17225–17235, November 2016.

[262] Wen Li, Linglang Zheng, Binghui Ge, Siqi Lin, Xinyue Zhang, Zhiwei Chen, Yunjie Chang, and Yanzhong Pei. Promoting SnTe as an eco-friendly solution for p-PbTe thermoelectric via band convergence and interstitial defects. *Advanced Materials*, 29(17):1605887, February 2017.

[263] Dou-Dou Liang, Zhen-Hua Ge, He-Zhang Li, Bo-Ping Zhang, and Fu Li. Enhanced thermoelectric property in superionic conductor Bi-doped $Cu_{1.8}S$. *Journal of Alloys and Compounds*, 708:169–174, June 2017.

[264] Plot Digitizer description. `http://plotdigitizer.sourceforge.net/`. Accessed: 2018-10-05.

[265] Byungki Ryu, Jaywan Chung, and SuDong Park. Thermoelectric efficiency has three degrees of freedom. *arXiv e-prints*, page arXiv:1810.11148, Oct 2018.

[266] Byungki Ryu, Jaywan Chung, and SuDong Park. General efficiency theory of thermoelectric conversion. *arXiv e-prints*, page arXiv:1910.11132, Oct 2019.

[267] P. Hohenberg and W. Kohn. Inhomogeneous electron gas. *Phys. Rev.*, 136:B864–B871, Nov 1964.

[268] W. Kohn and L. J. Sham. Self-consistent equations including exchange and correlation effects. *Phys. Rev.*, 140:A1133–A1138, Nov 1965.

[269] John P. Perdew, Kieron Burke, and Matthias Ernzerhof. Generalized gradient approximation made simple. *Phys. Rev. Lett.*, 77:3865–3868, Oct 1996.

[270] G. Kresse and J. Furthmüller. Efficient iterative schemes for ab initio total-energy calculations using a plane-wave basis set. *Phys. Rev. B*, 54:11169–11186, Oct 1996.

[271] P. E. Blöchl. Projector augmented-wave method. *Phys. Rev. B*, 50:17953–17979, Dec 1994.

[272] Byungki Ryu, Bong-Seo Kim, Ji Eun Lee, Sung-Jae Joo, Bok-Ki Min, HeeWoong Lee, Sudong Park, and Min-Wook Oh. Prediction of the band structures of Bi_2Te_3-related binary and Sb/Se-doped ternary thermoelectric materials. *Journal of the Korean Physical Society*, 68:115–120, Feb 2016.

[273] Byungki Ryu, Jaywan Chung, Eun-Ae Choi, Bong-Seo Kim, and Su-Dong Park. Thermoelectric power factor of Bi-Sb-Te and Bi-Te-Se alloys and doping strategy: First-principles study. *Journal of Alloys and Compounds*, 727:1067–1075, 2017.

[274] Georg K. H. Madsen and David J. Singh. BoltzTraP. a code for calculating band-structure dependent quantities. *Comput. Phys. Commun.*, 175:67–71, 2006.

[275] Atsushi Togo, Laurent Chaput, and Isao Tanaka. Distributions of phonon lifetimes in brillouin zones. *Phys. Rev. B*, 91:094306, Mar 2015.

[276] Byungki Ryu and Min-Wook Oh. Computational simulations of thermoelectric transport properties. *J. Korean Ceram. Soc.*, 53:273–281, May 2016.

[277] SciPy's `gaussian_filter1d` function description. `https://docs.scipy.org/doc/scipy/reference/generated/scipy.ndimage.gaussian\_filter1d.html`. Accessed: 2020-07-11.

(Jaywan Chung, Byungki Ryu, SuDong Park) Energy Conversion Research Center, Korea Electrotechnology Research Institute (KERI), Changwon-si, Gyeongsangnam-do, 51543 Republic of Korea

Email address: `jchung@keri.re.kr`
